# Clinical Impact of Digital Health Interventions with Educational Components for Patients with Heart Failure: A Systematic Review and Meta-Analysis

**DOI:** 10.3390/healthcare14162460

**Published:** 2026-08-09

**Authors:** Mohammad T. Alashqar, Yazan K. Barqawi, Milap C. Nahata

**Affiliations:** 1 Institute of Therapeutic Innovations and Outcomes, College of Pharmacy, The Ohio State University, Columbus, OH 43210, USA; alashqar.1@osu.edu; 2 AstraZeneca, Gaithersburg, MD 20878, USA; yazan.barqawi@astrazeneca.com; 3 College of Medicine, The Ohio State University, Columbus, OH 43210, USA

**Keywords:** heart failure, digital health education, clinical outcomes, systematic review, meta-analysis

## Abstract

**Background:** Heart failure (HF) is a major global health issue, with high morbidity, death, and frequent hospital readmissions. Digital health interventions with educational components may improve clinical outcomes in HF, but the variability observed across studies has not yet been assessed through systematic review and meta-analysis in this population. **Objective:** To examine the impact of digital health interventions containing an identifiable patient-education component on all-cause mortality, hospital readmissions, and health-related quality of life (HRQoL) in adults with HF. **Methods:** A systematic review and meta-analysis were conducted in accordance with the PRISMA 2020 standards. Randomized controlled trials (RCTs) published between January 2015 and June 2025 were identified using PubMed and manual searches. Studies including adults with HF who had received digital health interventions with educational component using mobile apps, web-based platforms, tele-education, or digital therapies were eligible for the research. Primary outcomes included all-cause mortality, hospital readmission, and HRQoL. Risk ratios (RR) and mean differences (MD) were aggregated using fixed- or random-effects models, depending on heterogeneity. **Results:** Twenty-six RCTs involving diverse international cohorts were included. Digital health with education interventions were associated with lower all-cause mortality (RR = 0.80; 95% CI: 0.66–0.97; *p* = 0.02). Hospital readmission risk was also lower in intervention groups (RR = 0.88; 95% CI: 0.79–0.99; *p* = 0.03) compared with standard of care (SoC). For heart-failure–specific HRQoL, KCCQ scores did not differ significantly between digital health with education and SoC (10 studies; 1528 participants; MD = 2.28; 95% CI: −1.61 to 6.18; *I*^2^ = 46%). MLHFQ scores favored intervention (seven studies; 3400 participants; directionally harmonized MD = 4.60; 95% CI: 0.69–8.52; *I*^2^ = 85%). **Conclusions:** Digital health interventions with educational components offer clinically significant advantages for patients with HF, decreasing mortality and readmissions while enhancing selected HRQoL metrics. These findings may support the integration of digital education into multidisciplinary HF care. Future studies should concentrate on standardizing intervention components, testing for long-term viability, and determining cost-effectiveness.

## 1. Introduction

Heart failure (HF) is a chronic, gradually worsening disease that impacts approximately 55 million people globally, with serious adverse outcomes for morbidity, mortality, and healthcare costs. The burden of HF continues to climb, driven by aging populations, improved survival following acute cardiac events, and the rising prevalence of comorbid conditions including hypertension, obesity and diabetes [1]. Despite breakthroughs in pharmacological medications and device-based interventions, substantial mortality, frequent hospital readmissions, poor quality of life, and suboptimal adherence to guideline-directed medical therapy continue to persist [2].

According to the latest estimates published with the Global Burden of Disease Study 2021, the age-standardized prevalence rate (ASPR) of HF is 676.7 per 100,000 population, with higher rates in males (760.8 per 100,000) than females (604 per 100,000). The burden is expected to rise even further by 2050, especially in low- and middle-income countries. Ischemic heart disease and hypertensive heart disease remain the predominant etiologies of HF worldwide [3].

Patient education and self-management interventions are critical components of comprehensive HF care, leading to optimized clinical outcomes, diminished hospitalizations, and improved quality of life. Traditional education methods consisting of printed materials and face-to-face counseling are often hindered by insufficient resources, inconsistent delivery, and variable patient engagement. In response, digital health education has emerged as a promising strategy to overcome these obstacles by utilizing technology to deliver scalable, personalized, and interactive support [4].

Digital health education should be distinguished from telemonitoring and remote disease management. Educational interventions primarily aim to improve patients’ knowledge, self-care skills, treatment adherence, and recognition of worsening symptoms. In contrast, telemonitoring involves the remote transmission and assessment of clinical information, while remote disease-management programs may include clinician alerts, treatment adjustment, and teleconsultations. Many contemporary digital interventions integrate education with one or more of these functions and are therefore best characterized as multicomponent digital health interventions with educational components.

Digital health education interventions cover an extensive variety of modalities, including mobile apps, telemonitoring systems, digital therapeutics and web-based platforms. These tools often include features such as clinician alerts, automated feedback and tailored educational modules [5]. Certain interventions incorporate artificial intelligence (AI) to augment personalization and predictive assistance. By combining educational content with real-time monitoring and clinical decision support, these technologies aim to empower patients, enhance adherence, and enable engagement in proactive clinical care [6,7].

Findings from randomized controlled trials (RCTs) demonstrate the efficacy of digital interventions to enhance outcomes in HF. The TIM-HF2 trial reported that remote patient management significantly reduced days missed caused by unexpected cardiovascular-related hospitalizations and all-cause death [8]. COPE trial, which proposed an empowerment-based self-care education program in a European cohort, improved patient knowledge, self-care behaviors, and proved cost-effective [2]. BEAT-HF trial found that structured telemonitoring minimized 180-day readmission rates, although it highlighted issues such as limited patient engagement and technology literacy. Care4Heart trial indicated an increase in quality of life and medication adherence with a mobile app–based educational intervention, though benefits were small and varied across subgroups, notably among older adults and those with poor digital literacy [9].

Despite these encouraging findings, several research gaps remain. Considerable heterogeneity exists in intervention types, duration, and intensity, making it difficult to determine which specific digital strategies are most effective [10]. Previous studies have generally assessed single outcomes, e.g., either hospital readmission or quality of life, rather than providing a comprehensive, multi-domain assessment [11]. Many trials are limited by relatively small cohorts and brief follow-up durations, or limited diversity in patient populations, which limit generalizability [12,13]. Furthermore, only a few meta-analyses have comprehensively evaluated the comparative effectiveness of different digital health education modalities using standardized clinical outcomes. Tang et al. assessed only 6MWD and LVEF and included trials from database inception, while Scholte et al. focused exclusively on all-cause mortality and hospitalization, analyzing studies published from 1996 to 2022 [14,15]. Notably, a marked increase in the number of digital health trials have been conducted in the past three years, reflecting major advances in technology and clinical integration, advances not captured in many earlier analyses.

To address these gaps, this systematic review and meta-analysis examined the clinical effects of digital health interventions containing an identifiable patient-education componenton all-cause mortality, hospital readmission rate, and HRQoL among adults with HF. Specifically, it investigated their effectiveness in improving major outcomes of all-cause mortality, readmission rates and HRQoL. By synthesizing existing evidence across multiple digital modalities, this study aimed to provide a comprehensive, reliable, and quantitative assessment of the overall effects of digital health educational interventions on key clinical outcomes among patients with HF. It also sought to inform clinical practice, guide implementation method, and identify priorities for future research with digital health education for HF care.

## 2. Methods

### 2.1. Study Design

This study was designed and conducted as a systematic review and meta-analysis following the Preferred Reporting Items for Systematic Reviews and Meta-Analyses (PRISMA 2020) guidelines [16]. All methodological aspects, including eligibility criteria, search strategy, data extraction, and synthesis, were determined a priori to enhance transparency and reproducibility. However, the review protocol was not prospectively registered in PROSPERO.

### 2.2. Eligibility Criteria

We included English-language studies indexed in PubMed between 1 January 2015, and 30 June 2025. This period coincided with the increasing availability and adoption of smartphones and mobile applications. For example, smartphone ownership among US adults increased from 35% in 2011 to 64% in 2015 [17]. Earlier HF mobile-health research predominantly evaluated older technologies, such as telephone calls and interactive voice-response systems, whereas newer applications increasingly support automated self-monitoring, symptom and medication tracking, reminders, personalized feedback, and communication with healthcare professionals [18]. Restricting the review to studies published from 2015 onward was therefore intended to reduce technological heterogeneity and enhance the applicability of the findings to contemporary HF care. Eligible studies were RCTs involving adult patients (age ≥ 18 years) diagnosed with HF, including reduced ejection fraction (HFrEF), preserved ejection fraction (HFpEF), or mildly reduced ejection fraction (HFmrEF). The interventions studied included a digital health intervention with educational components defined as interventions that delivered structured, patient-directed education through a digital platform, including mobile applications, web-based platforms, tablets, digital therapeutics, or telehealth systems. Eligible interventions could also incorporate telemonitoring, symptoms or biometric tracking, automated feedback, clinician alerts, teleconsultations, or medication-management support, provided that patient education was an explicitly identifiable component. Interventions based exclusively on monitoring or clinician-directed management without a patient-education component were excluded. Eligible studies measured at least one of the following outcomes: all-cause mortality, readmission rates and HRQoL.

The quantitative HRQoL synthesis was restricted to instruments developed specifically for HF: Kansas City Cardiomyopathy Questionnaire (KCCQ) and the Minnesota Living with HF Questionnaire (MLHFQ).

### 2.3. Search Strategy

A comprehensive electronic search of the PubMed and the Cochrane Central Register of Controlled Trials (CENTRAL) databases was conducted. The search approach combined Medical Subject Headings (MeSH), free-text keywords, and Boolean phrases. The complete search query is provided in the Appendix A. In brief, the search included terms related to HF, digital health education interventions, key clinical outcomes (all-cause mortality, readmission rates and HRQoL), and RCTs.

Additional searches of gray literature, including reference lists from relevant reviews and included studies, were conducted to identify articles not captured by the database search.

### 2.4. Study Selection, Data Extraction and Management

All records retrieved from the databases and manual searches were imported into Covidence systematic review software (Veritas Health Innovation, Melbourne, Australia). Covidence automatically identified and removed potential duplicate records using available bibliographic information, including titles, authors, publication years, and digital object identifiers. Potential duplicates were manually reviewed before exclusion.

Two independent reviewers screened titles and abstracts for relevance, followed by full-text evaluation to determine eligibility using predefined inclusion and exclusion criteria. Disagreements among reviewers were resolved by discussion or consultation with a third reviewer.

Two reviewers independently conducted data extraction using a standardized data extraction form. Data extracted included study information (author, year, country, and design), patient characteristics, sample size, type of intervention, comparator, duration, NYHA class, LVEF and outcome measures. The extracted outcomes included of all-cause mortality, readmission rates and HRQoL. Preprocessing algorithms were used to remove duplicates and standardize the data format and confirm internal consistency. The data were tabulated in Microsoft Excel to ensure accuracy before analysis.

### 2.5. Risk of Bias Assessment

The methodological quality of the included RCTs was assessed by the Cochrane Risk of Bias 2.0 (RoB 2.0) tool [19]. This tool evaluated five major domains: bias resulting from the randomization procedure, bias owing to variations from intended interventions, bias due to missing outcome data, bias in measurement of outcomes, and bias in selection of the reported results. Each domain was rated as a low risk of bias, some concerns, or high risk, with each study ultimately being quantified with an overall judgment. The assessments were performed by two independent reviewers, with any inconsistencies being resolved by consensus or by the engagement of a third reviewer.

### 2.6. Sensitivity Analyses

Leave-one-out influence analyses were performed for all outcomes to assess the influence of individual studies on the pooled effect estimates and to evaluate the robustness and stability of the meta-analysis results.

### 2.7. Publication Bias

Publication bias was initially assessed by visual inspection of funnel plots for outcomes including at least 10 studies. To provide a formal statistical assessment, Egger’s regression test and Begg’s rank correlation test were also performed for outcomes with sufficient numbers of studies (≥10), including all-cause mortality, hospital readmission, and HRQoL (KCCQ). A two-sided *p* value < 0.05 was considered indicative of potential publication bias.

### 2.8. Certainty of Evidence

The GRADE framework was followed to rate certainty of evidence across outcomes, considering risk of bias, inconsistency, indirectness, imprecision, and publication bias. The findings are summarized and presented.

### 2.9. Statistical Analysis

Meta-analyses were carried out using Review Manager (RevMan) version 5.3 software (Cochrane Collaboration, Copenhagen, Denmar). Pooled estimates of effect sizes were calculated as Risk Ratio (RR) for dichotomous outcomes and Mean Differences (MD) for continuous outcomes, using the Mantel–Haenszel method. All results were presented as two-sided 95% confidence intervals (CI). Between-study heterogeneity was quantified using Cochran’s Q test and *I*^2^ statistic. The *I*^2^ values were evaluated as follows: 0–25% indicated low heterogeneity, 26–50% moderate heterogeneity, 51–75% substantial heterogeneity, and >75% significant heterogeneity. A fixed-effects model was used when heterogeneity was low (*I*^2^ < 50%) and a random-effects model was employed when substantial heterogeneity was detected (*I*^2^ ≥ 50%). Potential publication bias was visually assessed through funnel plot asymmetry. A two-sided *p*-value of < 0.05 was considered statistically significant for all analyses.

KCCQ and MLHFQ outcomes were analyzed separately using raw mean differences because all studies within each instrument-specific analysis reported results on the same scale. The two instruments were not combined into an overall HRQoL estimate because they differ in their content, scoring systems, and score direction. KCCQ scores range from 0 to 100, with higher scores indicating better health status. Higher original MLHFQ scores indicate greater adverse effects of HF and poorer HRQoL; therefore, MLHFQ effect estimates were harmonized so that positive values represented better outcomes with digital health interventions with education. Random-effects models were used because clinical and methodological heterogeneity was anticipated.

## 3. Results

**Figure 1:**
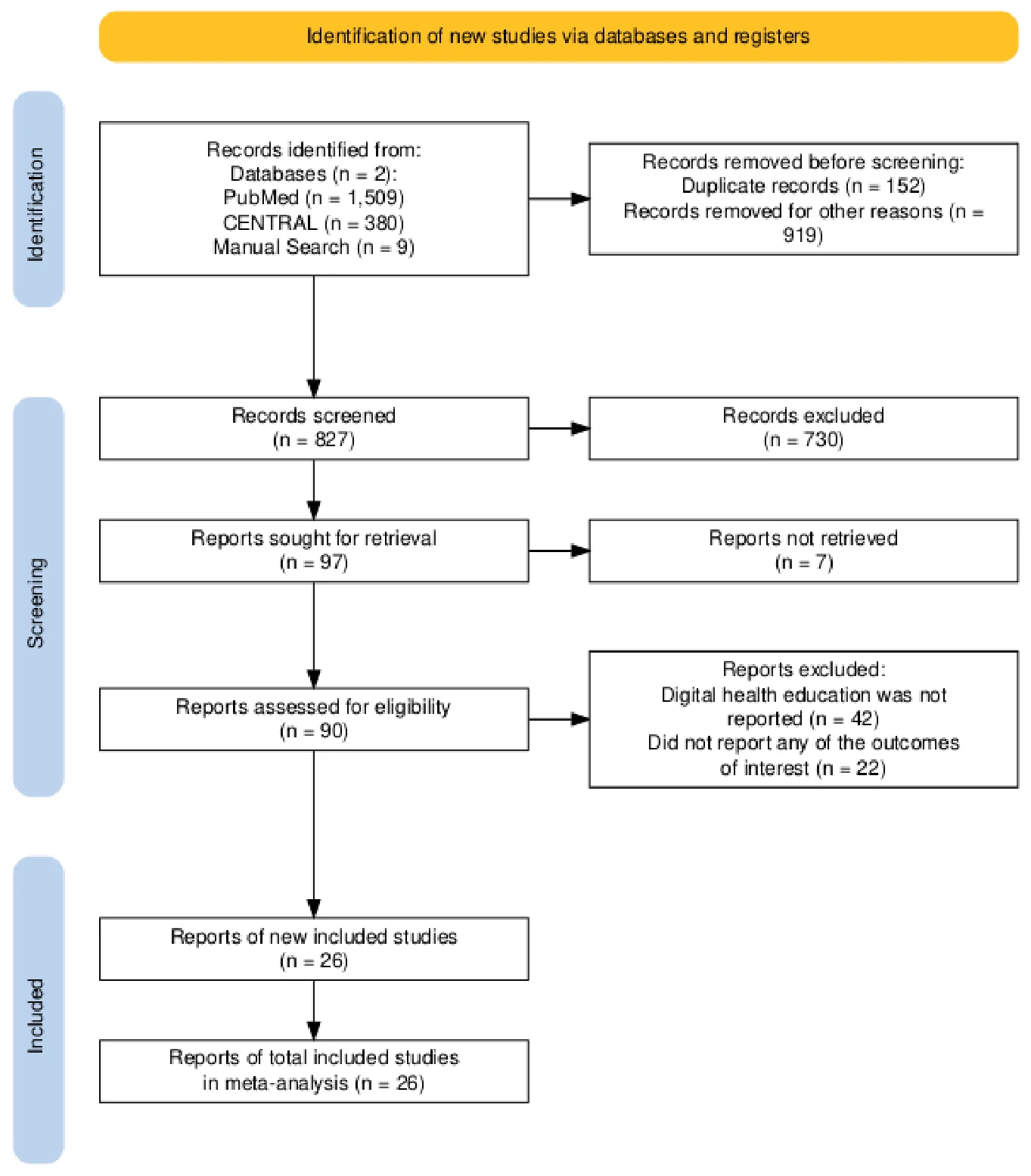
PRISMA flow diagram.

A total of 1898 records were identified: 1509 from PubMed, 380 from CENTRAL, and 9 through manual searching. Before screening, 152 duplicate records and 919 records were removed for other reasons. The remaining 827 records underwent title and abstract screening, of which 730 were excluded for being irrelevant, not RCTs, or not addressing HF. Of the 97 reports sought for retrieval, 7 could not be retrieved, leaving 90 reports for full-text eligibility assessment. Following a full-text review, 64 records were excluded because they did not report on digital health interventions with education or relevant outcomes (all-cause mortality, readmission rates and HRQoL). Ultimately, 26 studies were included in the review and meta-analysis [20,21,22,23,24,25,26,27,28,29,30,31,32,33,34,35,36,37,38,39,40,41,42,43,44,45] (Figure 1).

### 3.1. Characteristics of Included Studies

#### 3.1.1. Study Characteristics

The 26 RCTs included in this review enrolled a diverse group of participants, with sample sizes ranging from 18 to 1538, indicating both small pilot studies and large multicenter trials. The studies were conducted in many regions, including North America (USA, Canada, *n* = 11), Europe (France, Netherlands, Spain, Poland, Germany, Denmark, Portugal, *n* = 10), South America (Brazil, *n* = 1), and Asia (Iran, South Korea, *n* = 4), demonstrating international representation.

#### 3.1.2. Patient Characteristics

Participants were predominantly older adults, with reported mean ages ranging from 59 to 75 years, which is consistent with the population generally affected by HF. Most trials demonstrated a male predominance exceeding 60–70%, reflecting the limited inclusion of younger and female patients. The most frequently represented groups across studies were NYHA Class II and III, corresponding to patients with moderate symptom burdens. Severe HF (Class IV) was relatively less commonly represented, generally accounting for less than 10%, while mild cases (Class I) were reported in only a few trials. These findings suggest that digital health interventions with education were primarily targeted at patients with moderate HF severity. Most trials included patients with HFrEF (EF ≤ 40%), either exclusively or as the dominant subgroup. A few studies included mixed populations with HFmrEF (41–49%) and HFpEF (≥50%). Intervention periods varied substantially, ranging from 4 weeks to 18 months, allowing assessment of both short-term and long-term impacts (Table 1).

### 3.2. All-Cause Mortality

**Figure 2:**
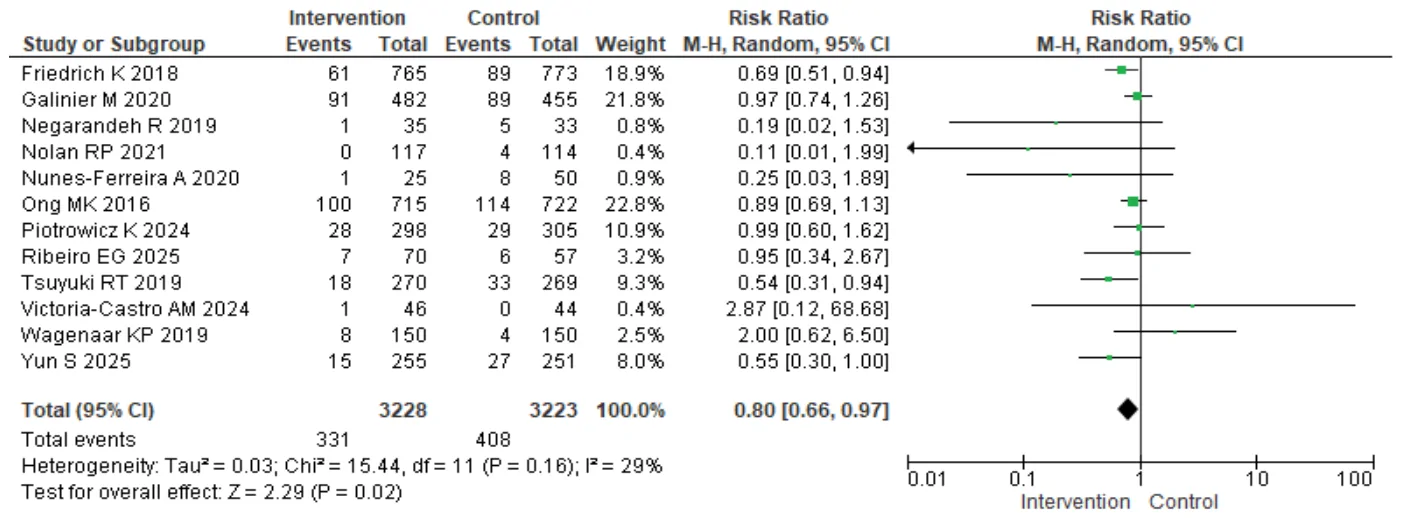
Forest plot of the effect of digital health interventions with educational components versus standard of care on all-cause mortality [22,23,24,25,26,27,31,37,39,42,43,44].

Figure 2 summarizes the meta-analysis of 12 studies evaluating all-cause mortality in patients receiving digital health with interventions with education compared with control, with the corresponding funnel plot presented in Supplementary Figure S1. The pooled analysis showed that the digital health interventions with education significantly reduced the risk of mortality compared with standard of care (SoC) (RR = 0.80; 95% CI: 0.66–0.97, *p* = 0.02). The heterogeneity was moderate (*I*^2^ = 29%), indicating that the combined estimate was reliable. Although a few individual studies did not reach statistical significance, this may be attributed to limited sample sizes or low event rates.

### 3.3. Hospital Readmission Rate

**Figure 3:**
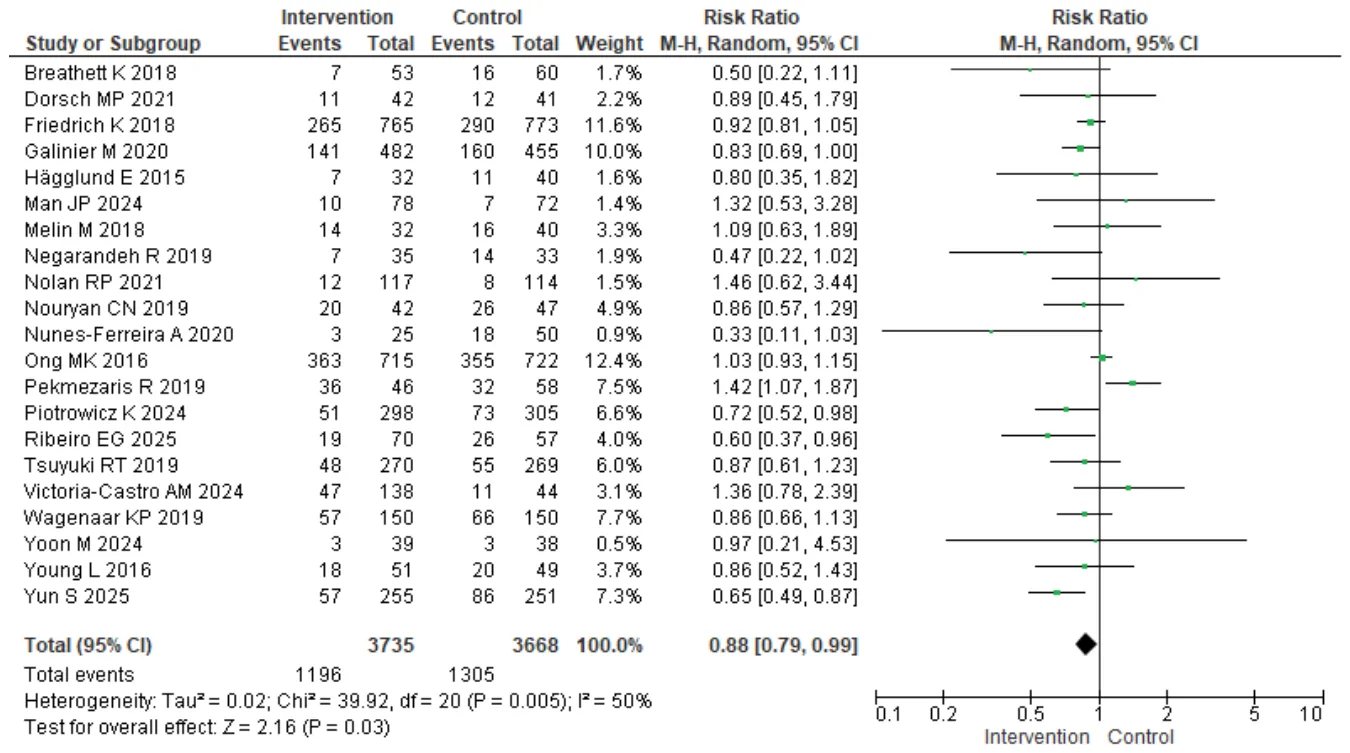
Forest plot of the effect of digital health interventions with educational components versus standard of care on hospital readmission [21,22,23,24,25,26,27,28,29,30,31,32,33,37,39,40,41,42,43,44,45].

The meta-analysis, illustrated in the forest plot, assessed the effect of digital health with education interventions versus SoC on hospital readmission, expressed as a risk ratio (RR). The pooled estimate indicated a significant reduction in readmission risk among participants receiving the intervention, with a pooled RR of 0.88 (95% CI: 0.79–0.99; *p* = 0.03). This corresponded to a 12% lower risk of readmission compared with SoC. The test for overall effect (Z = 2.16, *p* = 0.03) confirmed statistical significance. However, moderate heterogeneity was observed (Tau^2^ = 0.02; χ^2^ = 39.92, df = 20; *p* = 0.005; *I*^2^ = 50%), suggesting variability in effect sizes across studies, likely due to differences in patient populations, intervention components, and study methodologies. Despite this, the overall findings showed that digital health interventions with education were associated with a modest but statistically significant reduction in readmission risk, though the presence of heterogeneity warrants cautious interpretation (Figure 3, Supplementary Figure S2).

### 3.4. Health-Related Quality of Life

HRQoL was evaluated using two disease-specific instruments: KCCQ and MLHFQ. Separate meta-analyses were performed for each instrument because higher KCCQ scores indicate better health status, whereas lower MLHFQ scores indicate better HRQoL.

**Figure 4:**
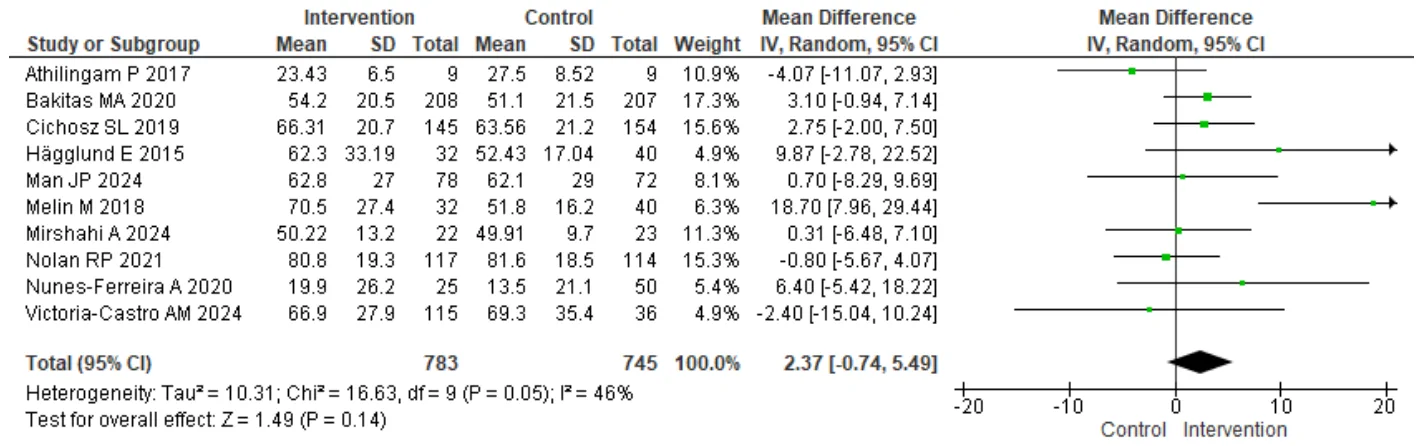
Forest plot of the effect of digital health interventions with educational components versus standard of care on health-related quality of life measured using the Kansas City Cardiomyopathy Questionnaire (KCCQ) [20,21,22,24,30,32,34,35,36,37].

The meta-analysis of 10 studies reporting KCCQ scores included 1528 participants (783 in the intervention groups and 745 in the control groups). Digital health interventions with education were not associated with a statistically significant improvement in KCCQ scores compared with SoC (MD = 2.37; 95% CI: −0.74 to 5.49; *p* = 0.14). Moderate heterogeneity was observed across studies (τ^2^ = 10.31; χ^2^ = 16.63; df = 9; *p* = 0.05; *I*^2^ = 46%) (Figure 4, Supplementary Figure S3).

**Figure 5:**
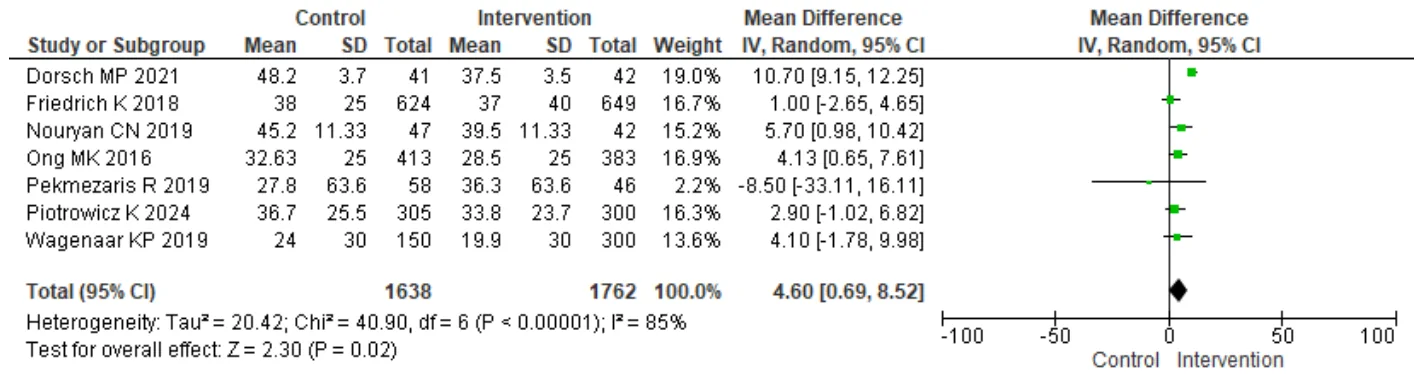
Forest plot of the effect of digital health interventions with educational components versus standard of care on health-related quality of life measured using the Minnesota Living with Heart Failure Questionnaire (MLHFQ) [27,28,31,33,39,41,42].

The meta-analysis of seven studies reporting MLHFQ scores included 3400 participants (1762 in the intervention groups and 1638 in the control groups). Digital health interventions with education were associated with a statistically significant improvement in MLHFQ scores compared with SoC (MD = 4.60; 95% CI: 0.69–8.52; *p* = 0.02), where a positive mean difference represented lower—and therefore better—MLHFQ scores in the intervention group. However, substantial heterogeneity was observed (τ^2^ = 20.42; χ^2^ = 40.90; df = 6; *p* < 0.00001; *I*^2^ = 85%) (Figure 5, Supplementary Figure S4).

### 3.5. Risk of Bias

Most of the studies showed low risk for the randomization procedure and outcome measurement, indicating robust allocation methods and objective, consistent assessments. Deviations from targeted interventions frequently raised concerns, owing mostly to open-label designs, with a few trials assessed as high risk due to unbalanced deviations or adherence issues. Missing outcome data varied from low risk to medium concern, with a few high-risk cases related to significant attrition and possible outcome-dependent missing. Selective reporting was typically of low risk, although some studies were high risk, often due to exploratory analyses that did not account for multiplicity. Overall, 15 studies were classified as low risk, 7 studies with some concerns, and 4 with high risk. Open-label designs, inadequate data, and unplanned sensitivity analyses were among the most common sources of bias (Supplementary Table S1).

### 3.6. Sensitivity Analyses

Leave-one-out sensitivity analyses showed that the magnitude and direction of the pooled estimates were generally consistent, although statistical significance varied after the exclusion of some studies (Table 2). For all-cause mortality, the pooled risk ratio ranged from 0.75 to 0.83, with low heterogeneity across analyses (*I*^2^ = 24–35%). The mortality benefit remained statistically significant after most individual studies were excluded; however, the confidence interval crossed or reached the null after removal of studies by Friedrich et al. (RR 0.82; 95% CI: 0.65–1.03), Tsuyuki et al. (RR 0.83; 95% CI: 0.69–1.01), and Yun et al. (RR 0.82; 95% CI: 0.68–1.00), indicating some sensitivity of the statistical significance to individual studies. For hospital readmission, the pooled risk ratio ranged from 0.86 to 0.91, with moderate heterogeneity across analyses (*I*^2^ = 37–53%). The reduction in hospital readmission remained statistically significant after exclusion of most individual studies. Exclusion of Pekmezaris et al. reduced heterogeneity from 50% to 37%, suggesting that this study was the largest contributor to between-study heterogeneity in the hospital readmission analysis. For KCCQ, all leave-one-out estimates remained nonsignificant, with pooled mean differences ranging from 1.37 to 3.08 and *I*^2^ values ranging from 0% to 52%; exclusion of Melin et al. reduced heterogeneity to 0%. For MLHFQ, the pooled mean difference remained positive in all analyses, ranging from 3.24 to 5.42. Statistical significance was retained after exclusion of most studies but was lost after removal of report by Nouryan et al. and was borderline after removal of Ong et al. Exclusion of Dorsch et al. report reduced heterogeneity from 85% to 0%, suggesting that this study was the primary contributor to heterogeneity in the MLHFQ analysis.

**Table 1:** Characteristics of the Included Randomized Controlled Studies Comparing Digital Health interventions with Education with Standard-of-Care Management.

Author, Year (Country)	Sample Size (*n*)	Intervention (*n*)	Control (*n*)	Mean Age, yr.	Sex Male (%)	HF Classification	Digital Health Education Intervention	Follow-Up Duration	Meta-Analysis Outcomes
Athilingam P et al., 2017 [35] (USA)	18	9	9	53	44	NYHA I: 5.6%, NYHA II: 61.1%, NYHA III: 33.3%	HeartMapp mobile app (daily use; features: symptom assessment, weight tracking, vital signs via Bluetooth chest strap, HF education, breathing exercises, walking, medication tracker, performance dashboard)	30 Days	HRQoL
Bakitas MA et al., 2020 [20] (USA)	415	208	207	63.8	53.3	96.9% NYHA III, 2.7% NYHA IV; LVEF < 40% in 43.3% (HFrEF), LVEF ≥ 40% in 56.3% (mix of HFmrEF/HFpEF)	ENABLE CHF-PC early palliative care: one in-person PC consult + six weekly nurse-coach telephonic sessions (20–40 min) + monthly follow-ups	16 Weeks	HRQoL
Breathett K et al., 2018 [29] (USA)	126	60	66	61	68	NR	NP education plus tablet application (interactive conditional logic program that flags patient questions to medical staff)	30 Days	Hospital Readmission
Choi EY et al., 2023 [38] (South Korea)	74	36	38	75	47	Experimental: NYHA I (16.7%), NYHA II (58.3%), NYHA III (19.4%), NYHA IV (5.6%) Control: NYHA I (5.3%), NYHA II (39.5%), NYHA III (39.5%), NYHAN IV (15.8%)	Heart Failure–SmartLife mobile app	3 Months	Hospital Readmission
Cichosz SL et al., 2018 [36] (Denmark)	299	145	154	70	80	NYHA II–IV	Telekit system (Samsung Galaxy Tab2 + BP monitor + scale); patients self-monitored vitals and answered disease-specific questionnaires; data assessed weekly by trained nurses; included educational component to empower patients in HF self-management	12 Months	HRQoL
Dorsch MP et al., 2021 [28] (USA)	83	42	41	60.2	65	NYHA I: 1 (2%), NYHA II: 15 (18%), NYHA III: 50 (60%), NYHA IV: 17 (20%); HFpEF: 35 (42%), HFrEF: remainder (58%)	ManageHF4Life mobile app with daily symptom tracking and health status indicator	12 Weeks	Hospital Readmission, HRQoL
Galinier M et al., 2020 [25] (France)	937	482	455	69.9	72.3	NYHA I: 6–7%, NYHA II: 43–44%, NYHA III: 38–41%, NYHA IV: 9–11; 59–61% had LVEF ≤ 40%	Daily telemonitoring: weight + 8 symptom questions transmitted via secure server Automated alerts reviewed by HF nurses, follow-up calls provided Educational component: information pack on HF + nurse calls every 3 weeks (medication adherence, recognition of decompensation signs, lifestyle counseling)	18 Months	All-Cause Mortality, Hospital Readmission,
Hägglund E et al., 2015 [32] (Sweden)	72	32	40	75	68	IG: NYHA II 38%, III 62% CG: NYHA II 18%, III 82% HFrEF: 54%, HFpEF: 36%, Mixed HFrEF/HFpEF: 10%	OPTILOGG^®^ consisting of a specialized software, a tablet computer (tablet) wirelessly connected to a weight scale, the tablet contained information about HF and lifestyle advice according to current guidelines. It also showed present dose of diuretic, changes in patient-measured weight and HRQoL over time	3 Months	Hospital Readmission, HRQoL
Koehler F et al., 2018 [39] (Germany)	1538	765	773	70	70	NYHA II (52%), NYHA III (47%), NYHA I/IV (<1%); HFrEF ≤ 40%: 43–45% HFmrEF 40–50%: 30–35%, HFpEF >50%: 22–25%	Remote patient management with daily monitoring (weight, BP, HR, ECG, SpO2), education, and 24/7 telemedical support	12 Months	All-Cause Mortality, Hospital Readmission, HRQoL
Man JP et al., 2024 [21] (Netherlands)	150	78	72	70	74	NYHA II: 74% (58/78 DC; 56/72 UC) NYHA III: 26% (20/78 DC; 15/72 UC); HFrEF only (LVEF ≤ 40%)	Digital consults (DC): remote, multifaceted strategy—digital data sharing from patient to clinic (medications, home BP/HR/weight, KCCQ), text-based e-learning for patients, and guideline-based recommendations summarized in the EHR before each consult; consults primarily via video/phone	12 Weeks	Hospital Readmission, HRQoL
Melin M et al., 2018 [30] (Sweden)	72	32	40	75	68	IG: NYHA II 38%, III 62% CG: NYHA II 18%, III 82%; HFrEF: 54% HFpEF: 36%, Mixed HFrEF/HFpEF: 10%	Tablet-based OPTILOGG system with educational modules and weight monitoring	6 Months	Hospital Readmission, HRQoL
Mirshahi A et al., 2024 [34] (Iran)	50	25	25	45–70 (Range)	60	NYHA II–III (inclusion criteria)	Nurse-led telehealth: six weekly live webinars (30–45 min) via SkyRoom + WhatsApp group content and peer case discussions; booklet provided; 6-week follow-up access	12 Weeks	HRQoL
Negarandeh R et al., 2019 [43] (Iran)	80	40	40	45–70 (Range)	60	NYHA II–III (inclusion criteria)	Tailored telephone-based telemonitoring with interactive education based on patient needs	8 Weeks	All-Cause Mortality, Hospital Readmission
Nolan RP et al., 2021 [22] (Canada)	231	117	114	59.5	78	NYHA I: 41–46%, NYHA II: 40–44% NYHA III: 13–15; LVEF ≤ 45% by inclusion	Automated e-counseling	12 Months	All-Cause Mortality, Hospital Readmission, HRQoL
Nouryan CN et al., 2019 [41] (USA)	89	42	47	83	32	NYHA I–II	Home telemonitoring station (LifeView, American TeleCare Inc.) with video, BP cuff, stethoscope, weight scale, pulse oximeter Daily uploads of vitals; weekly virtual nursing visit with review of vitals, lung sounds, and symptom counseling Personalized feedback, diet/exercise counselling, medication adherence checks	6 Months	Hospital Readmission, HRQoL
Nunes-Ferreira A et al., 2020 [37] (Portugal)	125	25	100	65.9	68	Mostly NYHA I–II (80–95%); some NYHA III (4–16%)	Telemonitoring with daily/3x weekly vitals, ECG, alerts, adherence scale	12 Months	All-Cause Mortality, Hospital Readmission, HRQoL
Ong MK et al., 2016 [42] (USA)	1437	715	722	73.5	53	NYHA I: 0.2–0.7%, NYHA II: 23–26%, NYHA III: 64–66%, NYHA IV: 10–11%	Telemonitoring + health coaching (daily vitals + nurse calls)	180 Days	All-Cause Mortality, Hospital Readmission, HRQoL
Pekmezaris R et al., 2019 [33] (USA)	104	46	58	59.9	59	30% NYHA II, 70% NYHA III, HFrEF 61%, HFmrEF 10%, HFpEF 29%	Telehealth self-monitoring (American TeleCare LifeView): daily vitals uploads (BP, SpO2, weight, pulse/heart rate) + weekly RN video visits; bilingual installation/training; remote stethoscope	90 Days	Hospital Readmission, HRQoL
Piotrowicz K et al., 2024 [27] (Poland)	605	300	305	67	79	NYHA I = 11%, NYHA II = 65%, NYHA III = 24%; 70% with LVEF ≤ 40%	Nurse-led non-invasive assessments with telemedical cardiologist support and structured education	12 Months	All-Cause Mortality, Hospital Readmission, HRQoL
Ribeiro EG et al., 2025 [23] (Brazil)	127	70	57	63.6	52	65% NYHA III/IV (IG 66%, CG 65%); 68% with LVEF (≤50%); 32% with LVEF (≥50%)	Multicomponent intervention: weekly nurse-led structured telephone support, self-care education via text messages, and access to cardiologist via teleconsultation	180 days	All-Cause Mortality, Hospital Readmission
Tsuyuki RT et al., 2019 [44] (Canada)	539	270	269	66	64	IG: NYHA III 38%, NYHA II 46%, NYHA IV 12% CG: NYHA III 45%, NYHA II 39%, NYHAIV 15% (Approx. 65% were NYHA III/IV overall)	A 20 min video with 3 simple self-care messages (avoid salt, daily weights, medication adherence), supplementary booklet + diary, 3 newsletters mailed at 2 weeks, 1, 3, and 5 months. Patients given DVD to rewatch at home	6 Months	All-Cause Mortality, Hospital Readmission
Victoria-Castro AM et al., 2024 [24] (USA)	182	138	44	61	62.1	Control: NYHA I (6.8%), NYHA II (38.6%), NYHA III (43.2%), Unknown (11.4%) Bodyport: NYHA I (4.4%), NYHA II (26.1%), NYHA III (69.6%); Conversa: NYHA I (8.7%), NYHA II (28.3%), NYHA III (47.8%), Unknown (15.2%) Noom: NYHA I (4.4%), NYHA II (26.1%), NYHA III (65.2%), Unknown (4.4%) HFrEF: 70.3% overall (128/182), HFpEF: 29.7% overall (54/182)	Digital education interventions: Bodyport: Smart cardiac scale measuring weight, impedance, HR + education modules, provider alerts Conversa: Automated conversational platform with HF education and symptom/BP monitoring Noom: Smartphone coaching app with behavior-change techniques, live coaching, support groups	90 Days	All-Cause Mortality, Hospital Readmission, HRQoL
Wagenaar KP et al., 2019 [31] (Netherlands)	450	300	150	67	74	NYHA I: 40–49%, NYHA II: 33–39%, NYHA III: 12–17%, NYHA IV: 5–12%; LVEF ≤ 40%: 70.4%	ESC/HFA website and e-health adjusted care pathway (EACP)	1 Year	All-Cause Mortality, Hospital Readmission, HRQoL
Yoon M et al., 2024 [40] (South Korea)	84	43	41	65.7	62	NYHA I: 11%, NYHA II: 60%, NYHA III: 26%, NYHA IV: 3%	Smartphone app with Bluetooth-connected devices (BP monitor, weight scale, body water analyzer), symptom tracking, education, and feedback system	4 Weeks	Hospital Readmission
Young L et al., 2016 [45] (USA)	100	51	49	70.2	36	NYHA II = 49%, NYHA III = 42%, NYHA IV = 9%	PATCH telephone-based coaching tailored to patient activation levels	3 Months	Hospital Readmission
Yun S et al., 2025 [26] (Spain)	506	255	251	73	59	NYHA I: 30 (12%) mHealth, 29 (12%) UC NYHA II: 160 (63%) mHealth, 164 (65%) UC NYHA III: 64 (25%) mHealth, 55 (22%) UC NYHA IV: 1 (<1%) mHealth, 3 (1%) UC; HFrEF (≤40%): 111 (44%) mHealth, 117 (47%) UC HFmrEF (41–49%): 37 (15%) mHealth, 29 (12%) UC HFpEF (≥50%): 107 (42%) mHealth, 105 (42%) UC	Mobile health (mHealth) combining telemonitoring and structured teleintervention via videoconference, daily biometric data and symptom questionnaire, and educational support	24 Weeks	All-Cause Mortality, Hospital Readmission

BP, blood pressure; CG, control group; ECG, electrocardiogram; EHR, electronic health record; ESC/HFA, European Society of Cardiology/Heart Failure Association; HF, heart failure; HFmrEF, heart failure with mildly reduced ejection fraction; HFpEF, heart failure with preserved ejection fraction; HFrEF, heart failure with reduced ejection fraction; HR, hazard ratio; HRQoL, health-related quality of life; IG, intervention group; KCCQ, Kansas City Cardiomyopathy Questionnaire; LVEF, left ventricular ejection fraction; NP, nurse practitioner; NR, not reported; NYHA, New York Heart Association class; PC, primary care; RN, registered nurse; SpO_2_, peripheral capillary oxygen saturation. Hägglund et al. [32] and Melin et al. [30] are separate reports from the same underlying OPTILOGG trial, presenting 3- and 6-month outcomes, respectively.

RR, risk ratio; CI, confidence interval; M-H, Mantel–Haenszel. Squares represent study-specific effect estimates weighted by study size, horizontal lines indicate 95% CIs, and the diamond represents the pooled effect estimate. The arrow indicates that the 95% CI extends beyond the displayed range of the x-axis. RR < 1 favors digital health interventions with educational components.

RR, risk ratio; CI, confidence interval; M-H, Mantel–Haenszel. A random-effects Mantel–Haenszel model was used to pool effect estimates. RR < 1 favors digital health interventions with educational components. Between-study heterogeneity was assessed using Cochran’s Q test and the *I*^2^ statistic. Squares represent study-specific effect estimates weighted by study size, horizontal lines indicate 95% confidence intervals, and the diamond represents the pooled effect estimate.

**Table 2:** Leave-one-out (Influence) sensitivity analyses for all-cause mortality, hospital readmission, and health-related quality of life outcomes.

	Study Removed	Pooled Estimate (%)	95% CI	*I*^2^ (%)
All-Cause Mortality	None (Original)	0.80	0.66, 0.97	29
	Friedrich K [39]	0.82	0.65, 1.03	28
	Galinier M [25]	0.75	0.60, 0.94	26
	Negarandeh R [43]	0.81	0.67, 0.97	26
	Nolan RP [22]	0.81	0.67, 0.97	26
	Nunes-Ferreira A [37]	0.81	0.66, 0.98	29
	Ong MK [42]	0.76	0.60, 0.98	33
	Piotrowicz K [27]	0.77	0.62, 0.96	33
	Ribeiro EG [23]	0.79	0.64, 0.97	35
	Tsuyuki RT [44]	0.83	0.69, 1.01	24
	Victoria-Castro AM [24]	0.79	0.65, 0.97	33
	Wagenaar KP [31]	0.78	0.65, 0.94	25
	Yun S [26]	0.82	0.68, 1.00	26
Hospital Readmission	None (Original)	0.88	0.79, 0.99	50
	Breathett K [29]	0.89	0.80, 1.00	49
	Dorsch MP [28]	0.88	0.79, 0.99	52
	Friedrich K [39]	0.87	0.77, 1.00	53
	Galinier M [25]	0.89	0.78, 1.00	51
	Hägglund E [32]	0.88	0.79, 0.99	52
	Man JP [21]	0.88	0.78, 0.98	52
	Melin M [30]	0.88	0.78, 0.98	52
	Negarandeh R [43]	0.89	0.80, 1.00	48
	Nolan RP [22]	0.88	0.78, 0.98	51
	Nouryan CN [41]	0.88	0.78, 0.99	52
	Nunes-Ferreira A [37]	0.89	0.80, 1.00	48
	Ong MK [42]	0.86	0.76, 0.98	43
	Pekmezaris R [33]	0.86	0.77, 0.95	37
	Piotrowicz K [27]	0.90	0.80, 1.01	49
	Ribeiro EG [23]	0.90	0.80, 1.01	48
	Tsuyuki RT [44]	0.88	0.78, 0.99	52
	Victoria-Castro AM [24]	0.87	0.78, 0.98	50
	Wagenaar KP [31]	0.88	0.78, 1.00	52
	Yoon M [40]	0.88	0.79, 0.99	52
	Young L [45]	0.88	0.79, 0.99	52
	Yun S [26]	0.91	0.81, 1.01	43
HRQoL—KCCQ	None (Original)	2.37	−0.74, 5.49	46
	Athilingam P [35]	3.08	−0.05, 6.22	40
	Bakitas MA [20]	2.39	−1.38, 6.17	51
	Cichosz SL [36]	2.45	−1.26, 6.16	52
	Hägglund E [32]	1.98	−1.19, 5.15	47
	Man JP [21]	2.59	−0.82, 6.00	52
	Melin M [30]	1.37	−0.76, 3.49	0
	Mirshahi A [34]	2.72	−0.79, 6.22	51
	Nolan RP [22]	2.99	−0.53, 6.51	47
	Nunes-Ferreira A [37]	2.17	−1.12, 5.46	50
	Victoria-Castro AM [24]	2.66	−0.62,5.94	50
HRQoL—MLHFQ	None (Original)	4.60	0.69, 8.52	85
	Dorsch MP [28]	3.24	1.41, 5.07	0
	Friedrich K [39]	5.42	1.49, 9.36	81
	Nouryan CN [41]	4.34	−0.20, 8.88	88
	Ong MK [42]	4.62	0.00, 9.25	86
	Pekmezaris R [33]	4.91	0.97, 8.84	87
	Piotrowicz K [27]	4.91	0.53, 9.30	86
	Wagenaar KP [31]	4.65	0.29, 9.01	87

CI, confidence interval; HRQoL, health-related quality of life; *I*^2^, inconsistency statistic; KCCQ, Kansas City Cardiomyopathy Questionnaire; MD, mean difference; MLHFQ, Minnesota Living with Heart Failure Questionnaire; RR, risk ratio. Leave-one-out (influence) sensitivity analyses were performed by sequentially omitting one study at a time and recalculating the pooled effect estimate and heterogeneity to evaluate the influence of individual studies on the overall results. For all-cause mortality, pooled effect estimates are presented as risk ratios (RR); for KCCQ and MLHFQ, pooled effect estimates are presented as mean differences (MD).

KCCQ, Kansas City Cardiomyopathy Questionnaire; MD, mean difference; CI, confidence interval; IV, inverse variance; SD, standard deviation. A random-effects inverse-variance model was used to pool effect estimates. Positive mean differences favor digital health interventions with educational components. Between-study heterogeneity was assessed using Cochran’s Q test and the *I*^2^ statistic. Squares represent study-specific effect estimates weighted by study size, horizontal lines indicate 95% confidence intervals, the arrow indicates that the 95% CI extends beyond the displayed range of the x-axis and the diamond represents the pooled effect estimate.

MLHFQ, Minnesota Living with Heart Failure Questionnaire; MD, mean difference; CI, confidence interval; IV, inverse variance; SD, standard deviation. A random-effects inverse-variance model was used to pool effect estimates. Mean differences were directionally harmonized so that positive values indicate better HRQoL and favor digital health interventions with educational components. Between-study heterogeneity was assessed using Cochran’s Q test and the *I*^2^ statistic. Squares represent study-specific effect estimates weighted by study size, horizontal lines indicate 95% confidence intervals, and the diamond represents the pooled effect estimate.

### 3.7. Publication Bias

Publication bias was evaluated using visual inspection of funnel plots together with Egger’s regression test and Begg’s rank correlation test (Table 3). For all-cause mortality, neither Egger’s regression test (*p* = 0.293) nor Begg’s test (*p* = 0.583) indicated evidence of publication bias. Similarly, no evidence of publication bias was detected for hospital readmission (Egger’s test *p* = 0.188; Begg’s test *p* = 0.856) or HRQoL assessed using the KCCQ (Egger’s test *p* = 0.393; Begg’s test *p* = 0.531). These statistical findings were consistent with the visual inspection of the funnel plots.

### 3.8. Certainty of Evidence

Table 4 summarizes the certainty of evidence using the GRADE approach. In the case of all-cause mortality, the certainty of evidence was assessed to be moderate because of issues associated with the risk of bias in some of the studies considered. Hospital readmission was also assessed to be moderate-certainty evidence for similar reasons. HRQoL evaluated using KCCQ was rated as moderate-certainty evidence. In contrast, the certainty of evidence for HRQoL assessed using MLHFQ was rated as very low because of concerns regarding risk of bias and very serious unexplained heterogeneity across studies. Consequently, confidence in the estimated effect of digital health interventions with educational components on MLHFQ-measured HRQoL was limited.

## 4. Discussion

This systematic review and meta-analysis demonstrated that digital health interventions with education meaningfully improved several clinically important outcomes in adults with HF. Across 26 RCTs, patients who received digitally delivered educational interventions experienced fewer hospital readmissions and lower all-cause mortality compared with those receiving usual SoC. These findings reinforce the growing recognition that digital platforms can provide timely, personalized, and accessible support to enhance self-management behaviors in this high-risk population.

Digital health interventions with education were associated with a 20% relative risk reduction in all-cause mortality, a clinically significant finding given the prior variability of mortality benefits in telemonitoring trials. For example, the Tele-HF experiment [46] failed to show a reduction in mortality, likely due to limited patient interaction and lack of personalized feedback. In contrast, interventions included in our study frequently incorporated interactive modules, clinician alerts, and tailored education, which may explain the observed survival benefit. These aspects are anticipated to improve adherence to guideline-directed therapy and promote early detection of decompensation.

The pooled analysis showed a 12–17% reduction in readmission risk, consistent with the central importance of self-care in preventing decompensation. Digital health interventions with education, whether mobile applications, web-based modules, tele-education, or multimodal digital therapies may have reduced readmissions by improving patients’ understanding of symptom monitoring, medication adherence, dietary practices, and daily weight tracking. These components are foundational to guideline-recommended management and may help patients recognize early signs of deterioration, enabling prompt medical evaluation and preventing avoidable admissions. Leave-one-out sensitivity analyses demonstrated that the direction of effect remained generally consistent, although the statistical significance became sensitive to removal of several individual studies. Although several studies were judged to be at high risk of bias, leave-one-out sensitivity analyses did not identify any single study as disproportionately influencing the overall findings.

The disease-specific HRQoL analyses produced different findings. Although both KCCQ and MLHFQ are validated heart-failure–specific instruments, they are not interchangeable. The KCCQ assesses symptoms, physical and social limitations, and quality of life, with higher scores indicating better health status. In contrast, the MLHFQ measures the adverse physical, emotional, and socioeconomic consequences of HF, with higher original scores indicating poorer HRQoL. The instruments also differ in recall periods, domain composition, and responsiveness. Comparative research suggests that KCCQ may correlate more strongly with baseline functional status, whereas MLHFQ may be more responsive to changes in functional capacity [47]. These differences, together with variation in intervention content, follow-up duration, and patient characteristics, may explain why improvement was observed with MLHFQ but not KCCQ. The substantial heterogeneity in the MLHFQ analysis further indicates that its pooled result should be interpreted cautiously.

Considerable clinical and methodological heterogeneity was present across the included trials. Study populations differed in HF phenotype, NYHA functional class, disease severity, age, comorbidities, and baseline risk. Interventions also varied substantially, ranging from relatively simple digitally delivered educational content to multicomponent disease-management programs combining education with symptom tracking, biometric monitoring, clinician alerts, teleconsultations, medication support, and continuous clinical feedback. Differences in intervention intensity, duration, comparator care, follow-up periods, and outcome definitions further limited comparability. Consequently, the pooled estimates should be interpreted as average effects across heterogeneous populations and intervention formats and should not be assumed to apply uniformly to every digital modality or HF subgroup. Although sensitivity analyses were conducted to examine the influence of outlying studies, reliable subgroup analyses according to intervention type, follow-up duration, HF phenotype, or NYHA class were not feasible because intervention components overlapped and subgroup-specific data were inconsistently reported. The certainty of evidence was moderate for mortality, hospital readmission, and HRQoL assessed using the KCCQ. In contrast, the certainty of evidence for HRQoL assessed using the MLHFQ was very low. Therefore, although the pooled MLHFQ analysis demonstrated a statistically significant improvement with digital health interventions, this finding should be interpreted cautiously and confirmed in future well-designed randomized controlled trials using standardized intervention strategies and outcome assessments. Formal statistical testing (Egger’s regression and Begg’s rank correlation) did not identify evidence of publication bias.

The economic value and implementation feasibility of these interventions also require further consideration. A recent systematic review of 27 economic evaluations found that 24 studies (89%) considered digital health interventions for HF cost-effective; however, results varied according to the intervention, analytical perspective, implementation costs, and assumptions regarding reductions in healthcare utilization [48]. Consequently, favorable clinical outcomes may not translate uniformly into economic value across healthcare systems. Successful implementation will also depend on patient digital literacy and access, sustained engagement, integration with existing clinical workflows and electronic health records, clinician capacity to review and respond to transmitted information, data security, reimbursement, and technical support. Future studies should therefore include trial-based economic evaluations and implementation outcomes to determine whether these interventions can be delivered effectively and equitably in routine HF care [49].

The included trials varied in sample size, intervention formats, and follow-up durations, which may have contributed to wider confidence intervals or reduced statistical power, particularly for mortality endpoints. Furthermore, the diversity of digital tools and educational frameworks could limit direct comparability across interventions. Despite these differences, the consistency of directionality across outcomes suggested a robust underlying effect, supporting the integration of digital education into HF care pathways.

Most included interventions combined patient education with telemonitoring, clinician feedback, symptom tracking, or remote disease management. Consequently, the independent contribution of education could not be isolated. Modality-specific subgroup analyses were not performed because intervention components overlapped substantially and the number of studies within distinct categories was insufficient to produce reliable subgroup estimates.

The review protocol was not prospectively registered in PROSPERO or another publicly accessible registry. Although the eligibility criteria, outcomes, and analytical methods were determined a priori, the absence of prospective registration limits external verification.

Overall, these findings underscore the clinical value of digital health interventions with education as a complementary strategy to the SoC provided by traditional HF management. As health systems increasingly adopt digital solutions, future research should focus on identifying which components of digital education are most effective, determining optimal intervention intensity, and evaluating long-term adherence. Additionally, implementation studies are needed to understand real-world feasibility, cost-effectiveness, and patient engagement across diverse populations.

## 5. Conclusions

This systematic review and meta-analysis demonstrated that digital health interventions with educational components for adults with HF were associated with clinically relevant benefits. Compared with SoC, these interventions reduced all-cause mortality by about 20%, and the hospital readmission rate by 12%. Digital health interventions with education may improve selected measures of heart-failure–specific HRQoL, although findings varied by instrument. A significant improvement was observed using the MLHFQ, whereas the KCCQ analysis did not demonstrate a statistically significant difference. However, these findings should be interpreted cautiously because of heterogeneity in intervention components, populations, follow-up periods, and outcome measurement. Furthermore, because most interventions combined education with telemonitoring, clinician feedback, or remote disease management, the observed benefits cannot be attributed to education alone. Digital health interventions with education may be considered as an adjunct to standard HF care within multidisciplinary care frameworks, providing scalable and effective support when applied with attention to patient selection, technology access, and sustained engagement; however, additional high-quality evidence is needed before routine implementation can be recommended. Future research should concentrate on defining the most effective intervention components, determining the durability of effects, and measuring cost-effectiveness across varied populations and healthcare settings.

## Figures and Tables

**Table 3:** Assessment of publication bias using Egger’s regression test and Begg’s rank correlation test.

Outcome	No. of Studies	Egger’s Regression Test	Begg’s Rank Correlation Test	Interpretation
All-cause mortality	12	Intercept = −0.617, *t* = −1.11, *p* = 0.293	*z* = −0.55, *p* = 0.583	No evidence of publication bias
Hospital readmission	21	Intercept = −0.654, *t* = −1.37, *p* = 0.188	*z* = 0.18, *p* = 0.856	No evidence of publication bias
HRQoL (KCCQ)	10	Intercept = 1.015, *t* = 0.90, *p* = 0.393	*z* = 0.63, *p* = 0.531	No evidence of publication bias

HRQoL, health-related quality of life; KCCQ, Kansas City Cardiomyopathy Questionnaire. Publication bias was assessed using Egger’s regression test and Begg’s rank correlation test for outcomes including at least 10 studies. A two-sided *p* value < 0.05 was considered indicative of potential publication bias.

**Table 4:** GRADE assessment of the certainty of evidence for the primary outcomes.

Outcome	No. of Studies	Risk of Bias	Inconsistency	Indirectness	Imprecision	Publication Bias	Certainty of Evidence
All-cause mortality	12 RCTs	Serious	Not serious (*I*^2^ = 29%)	Not serious	Not serious	Not serious	⨁⨁⨁◯ Moderate
Hospital readmission	21 RCTs	Serious	Not serious (I^2^ = 50%)	Not serious	Not serious	Not serious	⨁⨁⨁◯ Moderate
HRQoL (KCCQ)	10 RCTs	Serious	Not serious (*I*^2^ = 46%)	Not serious	Not serious	Not serious	⨁⨁⨁◯ Moderate
HRQoL (MLHFQ)	7 RCTs	Serious	Very serious (*I*^2^ = 85%)	Not serious	Not serious	Not serious	⨁◯◯◯ Very low

GRADE, Grading of Recommendations Assessment, Development and Evaluation; HRQoL, health-related quality of life; KCCQ, Kansas City Cardiomyopathy Questionnaire; MLHFQ, Minnesota Living with Heart Failure Questionnaire; RCT, randomized controlled trial. Certainty of evidence was assessed across the domains of risk of bias, inconsistency, indirectness, imprecision, and publication bias. GRADE certainty of evidence: ⨁⨁⨁⨁, high; ⨁⨁⨁◯, moderate; ⨁⨁◯◯, low; ⨁◯◯◯, very low.

## Data Availability

The original contributions presented in this study are included in the article and/or Supplementary Material. Further inquiries can be directed to the corresponding authors.

## Supplementary Materials

The following supporting information can be downloaded at https://www.mdpi.com/article/10.3390/healthcare14162460/s1, Figure S1: Funnel Plot for All-Cause Mortality; Figure S2: Funnel Plot for Readmission; Figure S3: Funnel Plot for Health-Related Quality of Life—KCCQ; Figure S4: Funnel Plot for Health-Related Quality of Life—MLHFQ; Table S1: Risk of Bias Assessment Using RoB 2.

## Appendix A

The completed search query:

(“Heart Failure” OR “heart failure” OR “cardiac failure” OR “congestive heart failure” OR HFrEF OR HFpEF OR HFmrEF) AND (“Digital Health” OR “digital health” OR mHealth OR telehealth OR telemedicine OR eHealth OR “mobile application*” OR “smartphone app*” OR “web-based” OR “online education” OR “digital education” OR “digital therapeutic*” OR “tele-education”) AND (“hospital readmission” OR “readmission” OR “mortality” OR “death” OR “quality of life” OR QoL) AND (“randomized controlled trial” OR “randomized controlled trial” OR RCT).
