# Supplementary material for: Clinical Impact of Digital Health Interventions with Educational Components for Patients with Heart Failure: A Systematic Review and Meta-Analysis
Source: Healthcare (Basel). 2026 Aug 9;14(16):2460. doi: 10.3390/healthcare14162460 (PMC13511761; doi:10.3390/healthcare14162460)
Supplement: Supplementary file 1 [file healthcare-14-02460-s001.zip › healthcare-4430320-supplementary.pdf]

Figure S1. Funnel Plot for All-Cause Mortality

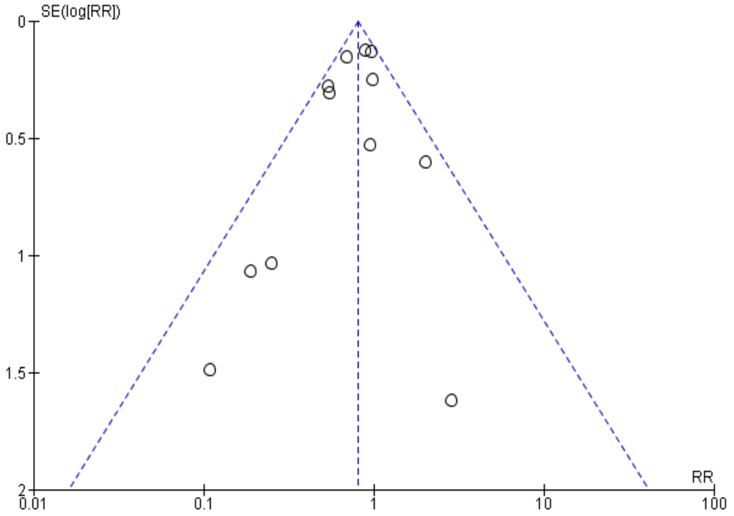

Figure S2. Funnel Plot for Readmission

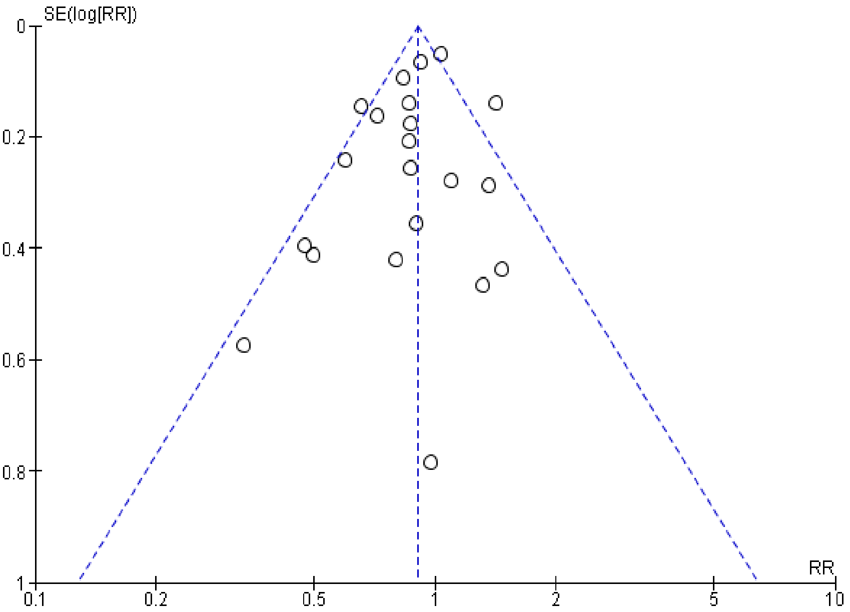

Figure S3. Funnel Plot for Health-related QOL- KCCQ

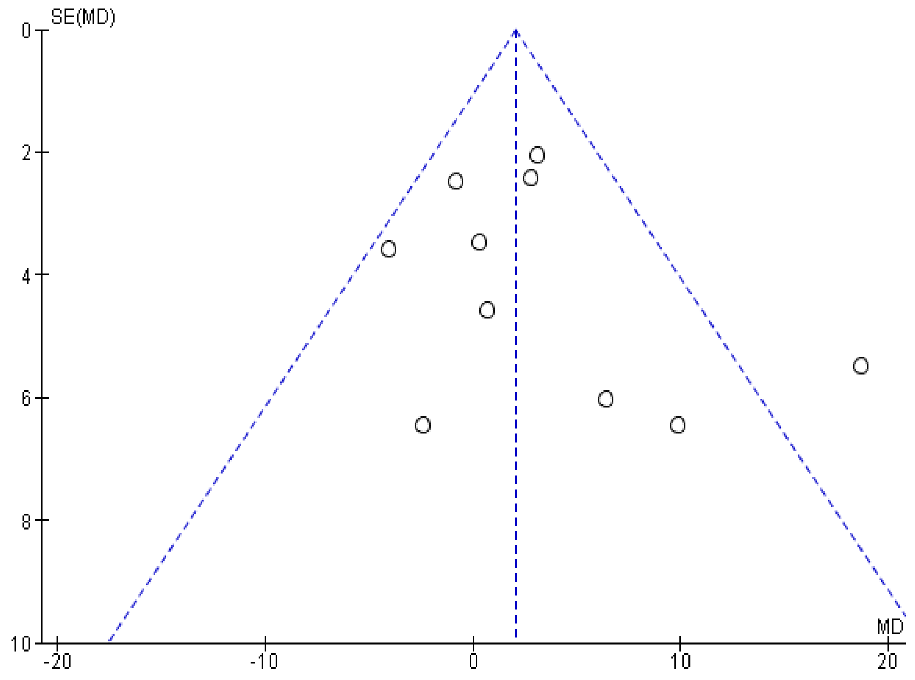

Figure S4. Funnel Plot for Health-related QOL- MLHFQ

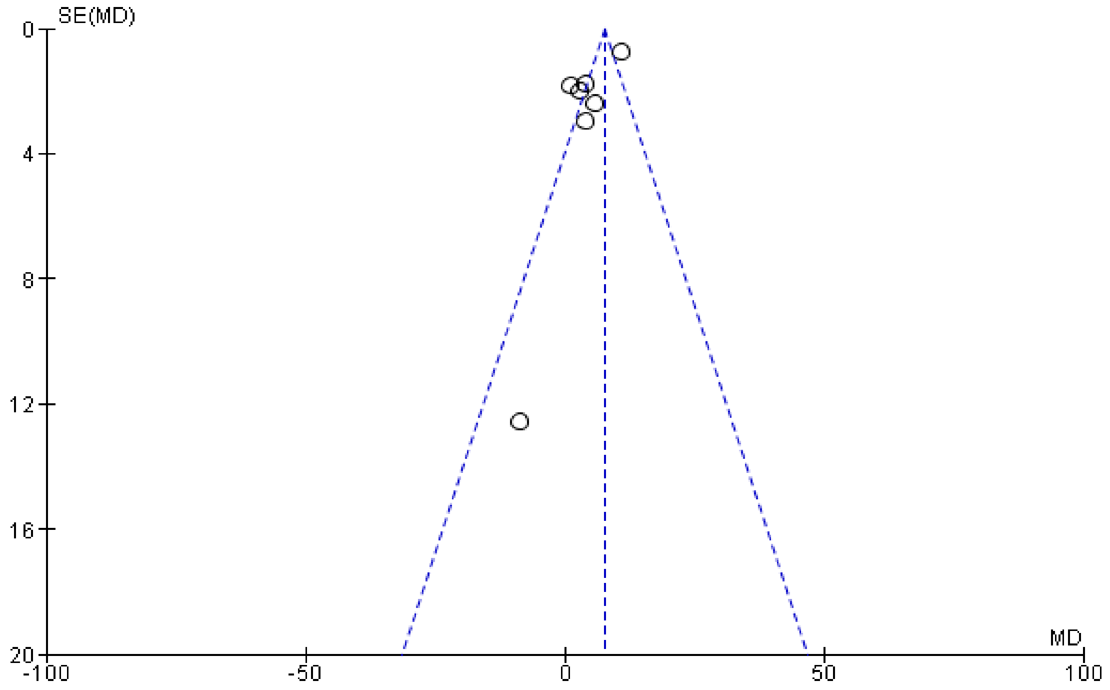

Table S1: Risk of Bias Assessment RoB-2

Author Name	Domain 1				Domain 2							
	Was the allocation sequence random?	Was the allocation sequence concealed until participants were enrolled and assigned to interventions?	Did baseline differences between intervention groups suggest a problem with the randomization process?	Risk-of-bias judgement:	Were participants aware of their assigned intervention during the trial?	Were carers and people delivering the interventions aware of participants' assigned intervention during the trial?	Were there deviations from the intended intervention that arose because of the trial context?	Were these deviations likely to have affected the outcome?	Were these deviations from intended intervention balanced between groups?	Was an appropriate analysis used to estimate the effect of assignment to intervention?	Was there potential for a substantial impact (on the result) of the failure to analyse participants in the group to which they were randomized?	Risk-of-bias judgement:
Bakitas MA	Yes - Randomization was computer-generated using REDCap with block size and stratification.	Yes- Allocation was concealed until after baseline data collection	No - Baseline characteristics were balanced	Low risk risk - The randomization process was adequately implemented, allocation was concealed, and baseline characteristics were comparable.	Yes-The trial was open-label; participants knew their group assignment.	Yes – Nurses and clinicians delivering the intervention were aware of group assignment.	Yes – Many participants missed the in-person consultation and/or did not complete all six sessions.	Yes – Low risker adherence may have contributed to the lack of significant improvement in primary outcomes	No – Deviations occurred only in the intervention arm.	Yes – ITT analysis were applied	No - All randomized participant were included in the ITT analysis	High risk – ITT analysis was used, the open-label design led to unbalanced deviations limited to the intervention arm
Man JP	Yes- Randomization was performed using a computerized tool (Castor EDC) with variable block sizes (2, 4, 6) and stratification by hospital and heart failure status (new-onset vs. established)	Yes- Allocation sequence was concealed during the enrollment process.	No- Baseline characteristics were well balanced between groups	Low risk risk - The randomization process was adequately implemented, allocation was concealed, and baseline characteristics were comparable.	Yes-The trial was open-label; participants knew their group assignment.	Yes - Clinicians needed to know the assignment to implement the digital consult strategy.	No- Major deviations from the intended intervention were not reported.	No- Deviations did not affect the outcomes.	Yes- Both groups folLow risked their respective protocols.	Yes-ITT analysis were used.	No - All randomized participant were included in the ITT analysis	High risk - open-label design, no major deviations occurred, both groups adhered to protocols, and ITT analysis included all randomized participants

Nolan RP	Yes- Randomization was done using an independent secure website.	Yes- Allocation was managed by an independent system	No - Baseline characteristics were similar between groups	Low risk risk - Randomization were adequate,allocation was managed and baseline balance was maintained.	No- Patients were blinded	No - Interventions were automated; research staff were blinded except one associate not involved in assessments.	No- Major deviations from the intended intervention were not reported.	No- Deviations did not affect the outcomes.	Yes - Both groups folLow risked their respective protocols.	Yes -ITT were applied	No - All randomized participant were included in the ITT analysis	Low risk- Open-label design, the trial maintained rigorous methodology, used ITT analysis, and had minimal deviations.
Ribeiro EG	Yes- Randomization was performed using simple randomization within site strata via a centralized web-based system	Yes- Allocation was automated and concealed via a secure web platform.	No- Baseline characteristics were balanced	Low risk risk - Randomization were adequate, allocation was concealed and baseline balance was maintained	Yes- The trial was open-label; participants knew their group assignment.	Yes- Nurse case managers and cardiologists involved in delivering the intervention were aware of group assignments.	Yes (minor). Some participants in the intervention group did not attend the initial visit.	No- Deviations did not affect the outcomes.	Yes- Both groups folLow risked their respective protocols.	Yes - ITT analysis were used.	No - All randomized participant were included in the ITT analysis	Low risk - Open-label design but deviations minimal.ITT analysis applied appropriately.
Victoria-Castro AM	Yes- Randomization was performed using a permuted block scheme created by an independent statistician."	Yes- Allocation sequence was concealed during enrollment.	No- Baseline characteristics were similar across groups.	Low risk risk - Randomization were adequate, allocation was concealed and baseline balance was maintained	Yes- The trial was open-label; participants knew their group assignment.	Yes-carers and people delivering the interventions were aware of participants' assigned	Yes- Some participants did not use the technology during folLow risk-up.	No- Deviations did not affect the outcomes.	Yes- Both groups folLow risked their respective protocols.	Yes - ITT analysis were used.	No - All randomized participant were included in the ITT analysis	Low risk- Open-label design but deviations minimal.ITT analysis applied appropriately.

Galinier M	Yes - Randomization was performed using a centralized system, ensuring a random allocation sequence.	Yes - Allocation sequence was concealed and centrally managed	No - Baseline characteristics were well balanced between groups.	Low risk risk - Randomization were adequate, allocation was concealed and baseline balance was maintained	Yes- The trial was open-label; participants knew their group assignment.	Yes - Nurses and investigators managing the telemonitoring system were aware of group assignments.	No - Major deviations from the intended intervention were not reported.	No - Deviations did not affect the outcomes.	Yes - Both groups folLow risked their respective protocols.	Yes - ITT analysis were used.	No - All randomized participant were included in the ITT analysis	Some concerns- Open-label design, deviations in intervention delivery, and potential influence of adherence introduce some risk of bias in the domain of deviations from intended interventions.
Yun S	Yes- Randomisation was done centrally via REDCap, ensuring a random sequence.	Yes- Allocation sequence was concealed and centrally managed	No- Baseline characteristics were well balanced between groups.	Low risk risk - Randomization were adequate, allocation was concealed and baseline balance was maintained	Yes- The trial was open-label; participants knew their group assignment.	Yes- Health-care providers were aware of group assignment	No- Major deviations from intended interventions were reported.	No - Deviations did not affect the outcomes.	Yes - Both groups folLow risked their respective protocols.	Yes- ITT analysis were used.	No - All randomized participant were included in the ITT analysis	Some concerns - open-label design and potential performance bias.
Piotrowicz K	Yes- Randomization was performed to assign patients to either the telecare or standard care group in this prospective, open-label, controlled study	Yes - Allocation sequence was concealed until participants were enrolled and assigned to interventions in the AMULET trial.	No- Baseline characteristics were similar across groups.	Low risk risk - Randomization were adequate, allocation was concealed and baseline balance was maintained	Yes- The trial was open-label; participants knew their group assignment.	Yes- Nurses and cardiologists knew the group assignments.	No - Major deviations from the intended intervention were not reported.	No- Deviations did not affect the outcomes.	Yes - Deviations were balanced between groups.	Yes- ITT analysis were used.	No - All randomized participant were included in the ITT analysis	Low risk- Open-label design may have influenced participant behavior, though intervention delivery was consistent.

Dorsch MP	Yes- Randomization was performed using the Trial Randomize application with a minimization approach to reduce covariate imbalances	Yes- Allocation concealment was likely ensured using the Trial Randomize application.	No- Baseline characteristics were similar between groups	Low risk risk - Randomization were adequate, allocation was concealed and baseline balance was maintained	Yes- The trial was open-label; participants knew their group assignment.	Yes- carers and people delivering the interventions were aware of participants' assigned intervention during the trial.	No - Major deviations from the intended intervention were not reported.	No- Deviations did not affect the outcomes.	Yes- Both groups folLow risked their respective protocols.	Yes- ITT analysis were used.	No - All randomized participant were included in the ITT analysis	Some Concerns- Open-label design meant participants and carers and people delivering the interventions aware of group assignments, which could have influenced behavior and engagement.
Breathett K	No- Randomization was performed to assign patients 1:1 to either nurse practitioner (NP) education plus tablet or NP education alone in this randomized controlled trial.	No- Allocation concealment method was not explicitly described.	No- Baseline characteristics were similar between groups	Low risk risk - Randomization were adequate, allocation was concealed and baseline balance was maintained	Yes- The trial was open-label; participants knew their group assignment.	Yes- Carers were likely aware of the intervention.	No - Major deviations from intended interventions were reported.	No - Deviations did not affect the outcomes.	Yes- Deviations were balanced between groups.	Yes- Mann–Whitney U tests were used	No- Participants were analyzed in their assigned groups.	Low risk - Open-label design, deviations in intervention delivery (especially discontinuation of NP education), and small sample size introduce some risk of bias, particularly in the domain of deviations from intended interventions.
Melin M	Yes – Randomization was performed by either standard care or standard care plus tablet-based intervention	No – Allocation concealment method not described.	No – Baseline characteristics were similar across groups.	Low risk risk - Randomization were adequate, allocation was concealed and baseline balance was maintained	Yes- The trial was open-label; participants knew their group assignment.	Yes – Carers were likely aware of the intervention	No – Major deviations from the intended intervention were not reported.	No – Deviations did not affect the outcomes.	Yes – Both groups folLow risked their respective protocols.	Yes – Mann-Whitney U test were used.	No- All randomized patients were included in the analysis.	Low risk - Open-label design, deviations in intervention delivery (especially discontinuation of NP education), and small sample size introduce some risk of bias, particularly in the domain of deviations from intended interventions.

Wagenaar KP	Yes - Randomization was performed using computerized block randomization.	Yes - Allocation sequence was concealed using computerized randomization.	No - Baseline characteristics were similar between groups	Low risk risk - Randomization were adequate, allocation was concealed and baseline balance was maintained	Yes- The trial was open-label; participants knew their group assignment.	Yes - Carers and nurses were aware of the intervention.	No - Major deviations from the intended intervention were not reported.	No - Deviations were not likely to affect the outcome.	Yes - Deviations were balanced between groups.	Yes - ANCOVA and COX analyses were used	No- All randomized patients were included in the analysis.	Low risk - Open-label design, unbalanced deviations and self-reported NYHA classification introduce some risk of bias, particularly in the domain of deviations from intended interventions.
Hägglund E	Yes – Randomization was performed by standard care	Yes – Allocation was concealed until after discharge and installation of the system.	No – Baseline characteristics were similar across groups.	Low risk risk - Randomization were adequate, allocation was concealed and baseline balance was maintained	Yes- The trial was open-label; participants knew their group assignment.	Yes – Carers and technical support staff were aware of intervention.	No – Major deviations from the intended intervention were not reported.	No – Deviations were not likely to affect the outcome.	Yes – Both groups folLow risked their respective protocols.	Yes – Mann-Whitney U test were used.	No- all randomized patients were included in the analysis.	Low risk – Open-label design, unbalanced deviations, and enhanced control group support introduce some risk of bias, particularly in the domain of deviations from intended interventions.
Pekmezaris R	Yes – Randomization was performed using a permuted block scheme stratification by NYHA class was used, ensuring equal representation across groups.	Yes – Allocation concealment was explicitly maintained	No – Baseline characteristics were similar across groups.	Low risk risk - Randomization were adequate, allocation was concealed and baseline balance was maintained	Yes- The trial was open-label; participants knew their group assignment.	Yes – carers and people delivering the interventions were aware of participants' assigned intervention during the trial.	Yes – Deviations occurred due to Low risk adherence in the TSM group	Yes - the deviations specifically the Low risk adherence to the telehealth self-monitoring (TSM) intervention were likely to have affected the outcome.	No – deviations from the intended intervention specifically Low risk adherence were not balanced between groups.	Yes – ITT analysis was performed.	No - All randomized participant were included in the ITT analysis	Some Concerns- Open-label design and potential influence of technology limitations introduce some risk of bias, particularly in the domain of deviations from intended interventions.

Mirshahi A	Yes – Randomization was performed using stratified block method with 1:1 assignment and block size of 4.	Yes – Allocation was concealed until participants were randomized and notified by the nurse interventionist.	No – Baseline characteristics were balanced between groups,	Low risk risk - Randomization were adequate, allocation was concealed and baseline balance was maintained	Yes- The trial was open-label; participants knew their group assignment.	Yes – Nurse interventionist and healthcare providers were aware of the assigned intervention.	No – Major deviations from the intended intervention were not reported.	No – Deviations were not likely to affect the outcome.	Yes – Both groups folLow risked their respective protocols.	Yes – ANCOVA test were used.	No- All randomized patients were included in the analysis.	Low risk – Open-label design, unbalanced deviations and cultural reluctance to discuss sensitive topics introduce some risk of bias, particularly in the domain of deviations from intended interventions.
Athilingam P	Yes - Randomization was performed using a permuted block design.	Yes - Allocation was concealed until participants were enrolled and assigned.	No - Baseline differences were not discussed	Low risk risk - Randomization were adequate, allocation was concealed and baseline balance was maintained	Yes- The trial was open-label; participants knew their group assignment.	Yes - Carers and interventionists were aware of group assignment.	No – Major deviations from the intended intervention were not reported.	No - Deviations were not likely to affect the outcome.	Yes - Both groups folLow risked their respective protocols.	Yes - t-tests and regression test were used	No- All randomized patients were included in the analysis.	Low risk - Open-label design, unbalanced deviations and small sample size introduce some risk of bias, particularly in the domain of deviations from intended interventions.
Simon L. C	Yes- Randomization was performed using pre-packed envelopes, ensuring a random allocation sequence.	Yes- Allocation concealment was maintained through the use of sealed envelopes.	No- Baseline characteristics were similar across groups	Low risk risk - Randomization were adequate, allocation was concealed and baseline balance was maintained	Yes- The trial was open-label; participants knew their group assignment.	Yes- Health care providers and nurses were aware of the intervention assignment.	No- Major deviations from the intended intervention were not reported.	No- Deviations were not likely to affect the outcome.	Yes- Deviations were balanced between groups.	Yes – ITT analysis was performed.	No - All randomized participant were included in the ITT analysis	Low risk risk - Open-label design, attrition, and unbalanced deviations introduce some risk of bias, particularly in the domain of deviations from intended interventions.

Afonso Nunes-Ferreira	Yes- Group allocation was performed using propensity matching, a statistical method used to simulate randomization in observational studies.	Yes- Allocation was concealed until participants were enrolled and assigned.	No- Baseline characteristics were similar across groups.	Low risk risk - Randomization were adequate, allocation was concealed and baseline balance was maintained	Yes- The trial was open-label; participants knew their group assignment.	Yes- carers and people delivering the interventions were aware of participants' assigned intervention during the trial.	No- Major deviations from the intended intervention were not reported.	No- Deviations were not likely to affect the outcome.	Yes- Both groups folLow risked their respective protocols.	Yes- ITT analysis were applied.	No - All randomized participant were included in the ITT analysis	Low risk- Open-label design, unbalanced deviations, and protocol adaptations introduce some risk of bias, particularly in the domain of deviations from intended interventions.
Eui-Young Choi	Yes – Randomization was performed using a computer system to assign participants to groups.	Yes – Allocation concealment was implied though not explicitly detailed.	Yes - Some baseline imbalances were present.	Some concerns- Lack of clarity on concealment and the presence of significant baseline imbalances	Yes- The trial was open-label; participants knew their group assignment.	Yes – Cardiac nurses, carers and people delivering the interventions were aware of participants' assigned intervention during the trial.	No – Major deviations from the intended intervention were not reported.	No – Deviations were not likely to affect the outcome.	Yes – Both groups folLow risked their respective protocols.	Yes – ANCOVA analysis were used	No- All randomized patients were included in the analysis.	Low risk- Open-label design, short intervention duration, and unbalanced deviations introduce some risk of bias, particularly in the domain of deviations from intended interventions.
Friedrich K	Yes – Randomisation was performed using Pocock's minimisation algorithm.	Yes – Allocation concealment was ensured via secure web-based system.	No – Baseline variables were balanced using minimisation algorithm.	Low risk risk - Randomization were adequate, allocation was concealed and baseline balance was maintained	Yes- The trial was open-label; participants knew their group assignment.	Yes – Carers and TMC staff were aware of group assignments.	No – Major deviations from the intended intervention were not reported.	No – Deviations not likely to affect outcomes.	Yes – Deviations were balanced between groups.	Yes – ANCOVA test were used.	No- All randomized patients were included in the analysis.	Low risk – Open-label design, potential deviations and adherence bias, the use of ITT analysis and structured protocols helps mitigate these risks.

Minjae Yoon	Yes – Randomization was performed using a web-based central randomization service with 1:1 allocation.	Yes – Allocation was concealed using a secure web-based system.	No – Baseline characteristics were well balanced between groups.	Low risk risk - Randomization were adequate, allocation was concealed and baseline balance was maintained	Yes- The trial was open-label; participants knew their group assignment.	Yes – Investigators interacting with patients were aware of group assignment.	No – Major deviations from the intended intervention were not reported.	No – Deviations were not likely to affect outcomes.	Yes – Deviations were balanced between groups.	Yes- ITT analysis were applied.	No - All randomized participant were included in the ITT analysis	Low risk – Open-label design, minor deviations , balanced exclusions and appropriate ITT analysis help mitigate these risks.
Christian N. Nouryan	Yes – Randomization was performed, but method not described.	No – Allocation concealment not reported.	No – Baseline characteristics were similar across groups.	Some concerns – Lack of detail on randomization and concealment.	Yes- The trial was open-label; participants knew their group assignment.	Yes – Nurses delivering intervention were aware of group assignment.	No – Major deviations from the intended intervention were not reported.	No – Deviations unlikely to affect outcomes.	Yes – Deviations were balanced between groups.	Yes- ITT analysis were applied.	No – All randomized participants were included in analysis.	Low risk – Open-label design and unbalanced adherence measurement raise concerns about performance and detection bias. However, ITT analysis and structured protocols help mitigate these risks.
Michael K. Ong	Yes – Randomization was performed using computerized block randomization.	Yes – Allocation concealment was likely ensured via computerized randomization, though not explicitly stated.	No – Baseline characteristics were similar across groups.	Low risk risk - Randomization were adequate, allocation was concealed and baseline balance was maintained	Yes- The trial was open-label; participants knew their group assignment.	Yes – Carers and nurses were aware of assigned interventions.	No – Major deviations from the intended intervention were not reported.	No – Deviations were not likely to affect outcomes.	Yes – Deviations were balanced between groups.	Yes- ITT analysis were applied.	No – All patients were analyzed in their assigned groups	Low risk – Open-label design and Low risk adherence in the intervention group raise concerns about performance and implementation bias. However, robust ITT analysis and large sample size help mitigate these risks.

Reza Negarandeh	Yes – Randomization was performed using fixed-size block randomization and allocation concealment via sealed envelopes.	Yes – Allocation was concealed using sealed envelopes until participants were enrolled.	No – Baseline characteristics were similar across groups.	Low risk risk - Randomization were adequate, allocation was concealed and baseline balance was maintained	Yes- The trial was open-label; participants knew their group assignment.	Yes – Nurses delivering the intervention were aware of group assignment.	No – Major deviations from the intended intervention were not reported..	No – Deviations were not likely to affect outcomes.	Yes – Deviations were balanced between groups.	Yes – ANCOVA, t-tests were used.	No – All patients were analyzed in their assigned groups	Low risk – Open-label design and minor loss to folLow risk-up raise concerns about performance and attrition bias. However, balanced group deviations and appropriate statistical adjustments mitigate these risks.
Ross T. Tsuyuki	Yes – Randomization was performed using a secure internet service with variable block design and stratification by site.	Yes – Allocation concealment was ensured via centralized randomization.	No – Baseline characteristics were similar across groups.	Low risk – Randomization and allocation concealment were appropriate and baseline balance was achieved.	Yes- The trial was open-label; participants knew their group assignment.	Yes – Coordinators delivering the intervention were aware of group assignments.	No – Major deviations from the intended intervention were not reported.	No – Deviations were not likely to affect outcomes.	Yes – Deviations were balanced between groups.	Yes – ITT analysis was used.	No – All randomized participants were analyzed in their assigned groups.	Low risk – Open-label design and variable adherence raise concerns about performance and implementation bias. However, balanced group deviations and robust ITT analysis mitigate these risks.

Lufei Young	Yes – Randomization used computer-generated sequence with variable block sizes and sealed envelopes.	Yes – Allocation concealment was ensured using sealed envelopes opened sequentially.	Yes- There are baseline imbalances that raise concerns	Some concerns- Presence of significant baseline imbalances	Yes- The trial was open-label; participants knew their group assignment.	Yes – Interventionists and care providers were aware of group assignment.	No – Major deviations from the intended intervention were not reported.	No – Deviations not likely to affect outcomes.	Yes – Deviations were balanced between groups.	Yes – ITT analysis were used.	No – All randomized participants were analyzed in their assigned groups.	Low risk – Open-label design, reliance on self-reported adherence, balanced group deviations and appropriate statistical methods mitigate these risks

Domain 4						Domain 5				Overall Risk of bias
Was the method of measuring the outcome inappropriate?	Could measurement or ascertainment of the outcome have differed between intervention groups?	Were outcome assessors aware of the intervention received by study participants?	Could assessment of the outcome have been influenced by knowledge of intervention received?	Is it likely that assessment of the outcome was influenced by knowledge of intervention received?	Risk-of-bias judgement:	Were the data that produced this result analysed in accordance with a pre-specified analysis plan that was finalized before unblinded outcome data were available for analysis?	Is the numerical result being assessed likely to have been selected, on the basis of the results, from		Risk-of-bias judgement:	Risk-of-bias judgement
							multiple eligible outcome measurements (e.g., scales, definitions, time points) within the	multiple eligible analyses of the data?		

							outcome domain?			
No – The study used validated and widely accepted tools for outcome measurement	No – Both groups had their outcomes assessed using the same tools and procedures.	No – Study was single-blind, with outcome assessors blinded to group allocation. Only participants, physicians, and interventionists were unblinded	No – Outcome assessors were blinded and the outcomes were self-reported using standardized tools, the risk of influence from knowledge of intervention is minimal.	No – Blinding of assessors and the use of validated self-report instruments, it is unlikely that outcome assessment was influenced by knowledge of group allocation	Low risk – Measurement methods were appropriate, consistent across groups, and assessors were blinded.	Yes-The data were analyzed according to a pre-specified analysis plan finalized before unblinded outcome data were available. The protocol and statistical plan were published and registered (ClinicalTrials.gov: NCT02505425)	Yes- The study includes multiple eligible outcome measurements	Yes- The study conducted multiple eligible analyses,	Some concern –primary analysis followed a pre-specified plan, the inclusion of unplanned sensitivity analyses suggests a possibility of selective reporting, though not enough to indicate high risk.	Low risk - The study follow risked a published protocol and statistical plan, used validated outcomes, and showed no signs of selective reporting or analysis bias.

No – The study used validated and widely accepted tools for outcome measurement	No – Both groups had their outcomes assessed using the same tools and procedures.	Yes- open-label, and participants and study staff were aware of group assignments	Yes-The trial was open-label, and clinicians were aware of group assignments, especially for the digital consult group, as they had to implement the intervention.	Yes- open-label design and self-reporting nature of the outcome introduce some risk of bias due to participant	Low risk- Outcome measurement was objective and protocol-driven, minimizing assessor influence.	Yes-The data were analyzed according to a pre-specified analysis plan finalized before unblinded outcome data were available. The protocol and statistical plan were published and registered (ClinicalTrials.gov: NCT05413447)	Yes- The study includes multiple eligible outcome measurements	Yes- The study conducted multiple eligible analyses,	Some concern –primary analysis followed a pre-specified plan, the inclusion of unplanned sensitivity analyses suggests a possibility of selective reporting, though not enough to indicate high risk.	Low risk - due to robust design, objective outcomes, and complete data. The trial's transparency and adherence to protocol support an overall
No – The study used validated and widely accepted tools for outcome measurement	No – Both groups had their outcomes assessed using the same tools and procedures.	No – The trial was double-blind: patients, research staff, and data analysts were blinded to	No- outcomes were self-reported and the trial was double-blind, there was no	No- The blinding procedures and use of automated, internet-based data collection	Low risk- Study was validated and applied uniformly across groups via automate	Yes-The data were analyzed according to a pre-specified analysis plan finalized before double blind outcome data were available. The protocol and statistical plan were published and	Yes- The study includes multiple eligible outcome measurements	Yes- The study conducted multiple eligible analyses,	Low risk-the trial followed a rigorous and pre-specified protocol, the use of	Low risk- The trial was well-designed with robust randomization, blinding, validated outcome measures, and pre-specified analysis. Minor

		group allocation.	opportunity for assessor bias to influence the results.	minimized any risk of bias due to knowledge of group assignment.	d online assessments. Blinding of assessors and automation minimized any influence on outcome measurement.	registered (NCT01864369)			multiple definitions for the primary outcome and exploratory analyses introduces a moderate risk of selective reporting.	concern about potential attrition bias, but impact is negligible.
No – The study used validated and widely accepted tools for outcome measurement	No – Both groups had their outcomes assessed using the same tools and procedures.	No- The study explicitly states that outcome assessment was blinded.	No- outcome assessors were blinded, there was no opportunity for their judgment to be influenced by knowledge of the intervention.	No- The blinding of assessors and the use of administrative data and predefined adjudication protocols, it is unlikely that outcome assessment was biased by interventi	Low risk - The trial used robust, objective outcome measures and blinded assessment, supporting a low risk of bias in the measurement of outcomes.	Yes -The data were analyzed according to a pre-specified analysis plan finalized before unblinded outcome data were available. The protocol and statistical plan were published and registered (NCT03149510)	Yes- The study includes multiple eligible outcome measurements	Yes- The study conducted multiple eligible analyses,	Some concern –primary analysis followed a pre-specified plan, the inclusion of unplanned sensitivity analyses suggests a possibility of selective reporting, though	Low risk- lack of participant blinding could influence behavior, but primary outcome (hospitalization) is objective and adjudicated blindly, reducing bias risk.

				on knowledge.					not enough to indicate high risk.	
No – The study used validated and widely accepted tools for outcome measurement	No – Both groups had their outcomes assessed using the same tools and procedures.	Yes- open-label, and participants and study staff were aware of group assignments	Yes-The trial was open-label, and clinicians were aware of group assignments, especially for the digital consult group, as they had to implement the intervention.	Yes- open-label design and self-reporting nature of the outcome introduce some risk of bias due to participant	Low risk- Despite self-report, validated tool and standardized process minimize bias.	Yes-The data were analyzed according to a pre-specified analysis plan finalized before unblinded outcome data were available. The protocol and statistical plan were published and registered (NCT04394754)	Yes- The study includes multiple eligible outcome measurements	Yes- The study conducted multiple eligible analyses,	Some concern –primary analysis followed a pre-specified plan, the inclusion of unplanned sensitivity analyses suggests a possibility of selective reporting, though not enough to indicate high risk.	Low risk- open-label design and self-reported primary outcome could introduce bias, though mitigated by validated tools and ITT analysis.

No – The study used validated and widely accepted tools for outcome measure ment	No – Both groups had their outcomes assessed using the same tools and procedur es.	No -The outcome assessors were explicitly stated to be blinded to group allocation, which is critical for reducing detection bias.	No - outcome s were objective , there was no opportun ity for bias due to knowled ge of group assignme nt	No - outcomes and blinded adjudicati on makes it highly unlikely that outcome assessme nt was influence d by knowledg e of the interventi on	Low risk - Outcome measure ment was appropria te and unbiased.	Yes- The data were analyzed according to a pre-specified analysis plan finalized before blinded outcome data were available. The protocol and statistical plan were published and registered .(NCT02068118)	Yes- The study includes multiple eligible outcome measure ments	Yes- The study conduc ted multipl e eligibl e analys es,	Some concern –primary analysis followed a pre-specified plan, the inclusion of unplanne d sensitivit y analyses suggests a possibilit y of selective reporting , though not enough to indicate high risk.	High risk- Randomization and allocation concealment were sound, missing data were minimal, and outcome measurement appears appropriate and unbiased.

No – The study used validated and widely accepted tools for outcome measurement	No – Both groups had their outcomes assessed using the same tools and procedures.	No -The clinical endpoint committee responsible for adjudicating outcomes was explicitly masked to treatment allocation	No- outcomes were objective there was no plausible mechanism for bias due to knowledge of group assignment.	No- outcomes, it is highly unlikely that outcome assessment was influenced by knowledge of the intervention.	Low risk -The HERMeS trial used robust outcome definitions, centralized and blinded adjudication, and objective clinical endpoints, supporting a low risk of bias in outcome measurement	Yes- The data were analyzed according to a pre-specified analysis plan finalized before unblinded outcome data were available. The protocol and statistical plan were published and registered (NCT03663907)	Yes- The study includes multiple eligible outcome measurements	Yes- The study conducted multiple eligible analyses,	Low risk -pre-specified protocol and masked endpoint adjudication. However, the inclusion of multiple secondary and exploratory analyses without correction for multiple comparisons introduces a moderate risk of selective reporting.	Low risk- Robust randomization and allocation concealment, high follow-up with ITT, objective outcomes with blinded adjudication, and minimal missing data together

No – The study used validated and widely accepted tools for outcome measurement	No – Both groups had their outcomes assessed using the same tools and procedures.	Yes- Open label design and self-reported surveys; participants were aware.	Yes-The trial was open-label, and clinicians were aware of group assignments, especially for the digital consult group, as they had to implement the intervention.	Yes- open-label design and self-reporting nature of the outcome introduce some risk of bias due to participant	Low risk- Despite self-report, validated tool and standardized process minimize bias.	Yes- The data were analyzed according to a pre-specified analysis plan finalized before blinded outcome data were available. The protocol and statistical plan were published and registered (ClinicalTrials.gov NCT03476590),	Yes- The study includes multiple eligible outcome measurements	Yes- The study conducted multiple eligible analyses,	Low risk-pre-specified protocol and appropriate statistical methods. However, the use of multiple outcome measures and exploratory analyses without correction for multiplicity introduces a moderate risk of selective reporting.	Low risk- Due to open-label design, missing data from folLow risk-up, and self-reported outcomes without blinding.

No – The study used validated and widely accepted tools for outcome measurement	No – Both groups had their outcomes assessed using the same tools and procedures.	Yes- Participants self-reported outcomes and were aware of their group.	Yes- Awareness of intervention may have influenced subjective responses.	Yes- Self-reported outcomes without blinding may have introduced bias.	Low risk- Self-reported questionnaires without blinding may have led to biased responses, especially in subjective measures like quality of life.	Yes - The data were analyzed according to a pre-specified analysis plan finalized before blinded outcome data were available. The protocol and statistical plan were published and registered (UTN U1111-1263-9802)	Yes- The study includes multiple eligible outcome measurements	Yes- The study conducted multiple eligible analyses,	Low risk-pre-specified protocol and appropriate statistical methods. However, the use of multiple outcome measures and exploratory analyses without correction for multiplicity introduces a moderate risk of selective reporting.	Some Concerns -Open-label design and self-reported outcomes may have introduced bias, and missing folLow risk-up data could affect the reliability of subjective measures.

No – The study used validated and widely accepted tools for outcome measurement	No – Both groups had their outcomes assessed using the same tools and procedures.	Yes- Open label design and self-reported surveys; participants were aware.	Yes- The trial was open-label, and clinicians were aware of group assignments, especially for the digital consult group, as they had to implement the intervention.	Yes- open-label design and self-reporting nature of the outcome introduce some risk of bias due to participant	Low risk- Despite self-report, validated tool and standardized process minimize bias.	Yes- Study folLow risked a clear protocol, used pre-specified outcomes and statistical methods, and transparently reported all analyses. There is no indication of selective reporting or data-driven analysis choices.	Yes- The study includes multiple eligible outcome measurements	Yes- The study conducted multiple eligible analyses,	Low risk- study folLow risked a clear protocol, used pre-specified outcomes and statistical methods, and transparently reported all analyses. There is no indication of selective reporting or data-driven analysis choices.	High risk- Randomization/ baseline balance were acceptable and outcomes used validated tools with prespecified analyses. However, outcome-dependent missingness, open-label self-reported endpoints, and intervention performance bias

No – The study used validated and widely accepted tools for outcome measurement	No – Both groups had their outcomes assessed using the same tools and procedures.	Yes- Open label design and self-reported surveys; participants were aware.	Yes-The trial was open-label, and clinicians were aware of group assignments, especially for the digital consult group, as they had to implement the intervention.	Yes- open-label design and self-reporting nature of the outcome introduce some risk of bias due to participant	Low risk- Despite self-report, validated tool and standardized process minimize bias.	Yes- The study folLow risked a pre-specified protocol, was approved by an ethics board (Dnr: 2012/2176-31/1), and used intention-to-treat analysis with clearly defined endpoints and statistical methods.	Yes- The study includes multiple eligible outcome measurements	Yes- The study conducted multiple eligible analyses,	Low risk- study folLow risked a clear protocol, used pre-specified outcomes and statistical methods, and transparently reported all analyses. There is no indication of selective reporting or data-driven analysis choices.	High risk- Randomized trial but allocation concealment not described, open-label with self-reported outcomes, and not all randomized patients included in the analysis raise performance/analysis bias concerns.

No – The study used validated and widely accepted tools for outcome measurement	No – Both groups had their outcomes assessed using the same tools and procedures.	Yes- Open label design and self-reported surveys; participants were aware.	Yes-The trial was open-label, and clinicians were aware of group assignments, especially for the digital consult group, as they had to implement the intervention.	Yes- open-label design and self-reporting nature of the outcome introduce some risk of bias due to participant	Low risk- Despite self-report, validated tool and standardized process minimize bias.	Yes- The data were analyzed according to a pre-specified analysis plan finalized before blinded outcome data were available. The protocol and statistical plan were published and registered (ClinicalTrials.gov ID NCT01755988)	Yes- The study includes multiple eligible outcome measurements	Yes- The study conducted multiple eligible analyses,	Low risk - Study folLow risked a registered protocol, used clearly defined and pre-specified outcomes, and transparently reported all analyses. There is no indication of selective reporting or data-driven analysis choices.	Low risk- Due to open-label design, missing data from folLow risk-up, and self-reported outcomes without blinding.

No – The study used validated and widely accepted tools for outcome measurement	No – Both groups had their outcomes assessed using the same tools and procedures.	Yes- Open label design and self-reported surveys; participants were aware.	Yes-The trial was open-label, and clinicians were aware of group assignments, especially for the digital consult group, as they had to implement the intervention.	Yes- open-label design and self-reporting nature of the outcome introduce some risk of bias due to participant	Low risk- Despite self-report, validated tool and standardized process minimize bias.	Yes- Study folLow risked a pre-specified protocol, was approved by the Regional Ethical Review Board (Dnr: 2012/2176-31/1), and used defined endpoints and statistical methods for analysis	Yes- The study includes multiple eligible outcome measurements	Yes- The study conducted multiple eligible analyses,	Low risk – study folLow risked a clear protocol, used pre-specified outcomes and statistical methods, and transparently reported all analyses. There is no indication of selective reporting or data-driven analysis choices.	Low risk – Adequate randomization with concealed allocation, baseline balance, validated outcome tools, and high follow-up/modified ITT support

No – The study used validated and widely accepted tools for outcome measurement	No – Both groups had their outcomes assessed using the same tools and procedures.	No -The outcome assessors were explicitly stated to be blinded to group allocation, which is critical for reducing detection bias.	No - outcomes were objective, there was no opportunity for bias due to knowledge of group assignment	No - outcomes and blinded adjudication makes it highly unlikely that outcome assessment was influenced by knowledge of the intervention	Low risk - Outcome measurement was appropriate and unbiased.	Yes- The data were analyzed according to a pre-specified analysis plan finalized before blinded outcome data were available. The protocol and statistical plan were published and registered (ClinicalTrials.gov ID: NCT02196922),	Yes- The study includes multiple eligible outcome measurements	Yes- The study conducted multiple eligible analyses,	Low risk -pre-specified protocol and appropriate statistical methods. However, the inclusion of multiple outcome measures and exploratory post hoc analyses without correction for multiplicity introduces a moderate risk of selective reporting.	Low risk – Overall well-conducted study with some concerns in adherence and intervention deviations.

No – The study used validated and widely accepted tools for outcome measurement	No – Both groups had their outcomes assessed using the same tools and procedures.	No- Outcome assessor and statistician were blinded to group allocation, reducing the risk of detection bias	No – Outcome assessors were blinded and the tools used were standardized and self-administered, it is unlikely that knowledge of group assignment influenced the assessment.	No – Outcomes were assessed by a blinded assessor using validated questionnaires and hospital records, minimizing the risk of bias from knowledge of intervention allocation.	Low risk - Outcome measurement was appropriate and unbiased.	Yes- The data were analyzed according to a pre-specified analysis plan finalized before blinded outcome data were available. The protocol and statistical plan were published and registered (IRCT20100725004443N29), approved by an ethics board	Yes- The study includes multiple eligible outcome measurements	Yes- The study conducted multiple eligible analyses,	Low risk - Study folLow risked a registered protocol, used clearly defined and pre-specified outcomes, and transparently reported all analyses. There is no indication of selective reporting or data-driven analysis choices.	Some concerns- Well-designed feasibility trial, but lack of blinding and moderate adherence issues raise some concerns. Missing data and outcome measurement were handled properly

No – The study used validated and widely accepted tools for outcome measurement	No – Both groups had their outcomes assessed using the same tools and procedures.	Yes- Open label design and self-reported surveys; participants were aware.	Yes-The trial was open-label, and clinicians were aware of group assignments, especially for the digital consult group, as they had to implement the intervention.	Yes- open-label design and self-reporting nature of the outcome introduce some risk of bias due to participant	Low risk- Despite self-report, validated tool and standardized process minimize bias.	No - Study does not mention a pre-specified analysis plan finalized before unblinded outcome data were available. It was a pilot feasibility trial with flexible exploratory analysis.	Yes- The study includes multiple eligible outcome measurements	Yes- The study conducted multiple eligible analyses,	Low risk - study folLow risked a clear protocol, used pre-specified outcomes and statistical methods, and transparently reported all analyses. There is no indication of selective reporting or data-driven analysis choices.	Low risk- Due to open-label design, missing data from folLow risk-up, and self-reported outcomes without blinding.

No – The study used validated and widely accepted tools for outcome measurement	No – Both groups had their outcomes assessed using the same tools and procedures.	No- Outcome assessor and statistician were blinded to group allocation, reducing the risk of detection bias	No – Outcome assessors were blinded and the tools used were standardized and self-administered, it is unlikely that knowledge of group assignment influenced the assessment.	No – Outcomes were assessed by a blinded assessor using validated questionnaires and hospital records, minimizing the risk of bias from knowledge of intervention allocation.	Low risk - Outcome measurement was appropriate and unbiased.	Yes- The data were analyzed according to a pre-specified analysis plan finalized before blinded outcome data were available. The protocol and statistical plan were published and registered (ClinicalTrials.gov ID: NCT02860013),	Yes- The study includes multiple eligible outcome measurements	Yes- The study conducted multiple eligible analyses,	Low risk - Study folLow risked a registered protocol, used clearly defined and pre-specified outcomes, and transparently reported all analyses. There is no indication of selective reporting or data-driven analysis choices.	Some concerns- While most domains show Low risk risk , imbalance in marital status and lack of blinding raise minor concerns.

No – The study used validated and widely accepted tools for outcome measurement	No – Both groups had their outcomes assessed using the same tools and procedures.	No- Outcome assessor and statistician were blinded to group allocation, reducing the risk of detection bias	No – Outcome assessors were blinded and the tools used were standardized and self-administered, it is unlikely that knowledge of group assignment influenced the assessment.	No – Outcomes were assessed by a blinded assessor using validated questionnaires and hospital records, minimizing the risk of bias from knowledge of intervention allocation.	Low risk - Outcome measurement was appropriate and unbiased.	Yes- Study was prospectively designed, approved by an ethics committee.	Yes- The study includes multiple eligible outcome measurements	Yes- The study conducted multiple eligible analyses,	Low risk - study folLow risked a clear protocol, used pre-specified outcomes and statistical methods, and transparently reported all analyses. There is no indication of selective reporting or data-driven analysis choices.	Low risk- Study design, execution, and analysis were robust with minimal bias.

No – The study used validated and widely accepted tools for outcome measurement	No – Both groups had their outcomes assessed using the same tools and procedures.	No- Outcome assessor and statistician were blinded to group allocation, reducing the risk of detection bias	No – Outcome assessors were blinded and the tools used were standardized and self-administered, it is unlikely that knowledge of group assignment influenced the assessment.	No – Outcomes were assessed by a blinded assessor using validated questionnaires and hospital records, minimizing the risk of bias from knowledge of intervention allocation.	Low risk - Outcome measurement was appropriate and unbiased.	Yes- Study was prospectively registered, approved by IRBs and folLow risked a pre-specified protocol with defined outcomes and statistical methods	Yes- The study includes multiple eligible outcome measurements	Yes- The study conducted multiple eligible analyses,	Low risk - Study folLow risked a registered protocol, used clearly defined and pre-specified outcomes, and transparently reported all analyses. There is no indication of selective reporting or data-driven analysis choices.	Some concerns – While the study was well-conducted overall, concerns remain regarding baseline imbalances, lack of blinding, and potential selective reporting.

No – The study used validated and widely accepted tools for outcome measure ment	No – Both groups had their outcomes assessed using the same tools and procedur es.	No -The outcome assessors were explicitly stated to be blinded to group allocation, which is critical for reducing detection bias.	No - outcome s were objective , there was no opportun ity for bias due to knowled ge of group assignme nt	No - outcomes and blinded adjudicati on makes it highly unlikely that outcome assessme nt was influence d by knowledg e of the interventi on	Low risk - Outcome measure ment was appropria te and unbiased.	Yes- The data were analyzed according to a pre-specified analysis plan finalized before blinded outcome data were available. The protocol and statistical plan were published and registered (ClinicalTrials.gov ID: NCT01878630)	Yes- The study includes multiple eligible outcome measure ments	Yes- The study conduc ted multipl e eligibl e analys es,	Low risk - Study folLow risked a registere d protocol, used clearly defined and pre-specified outcome s, and transpare ntly reported all analyses. There is no indicatio n of selective reporting or data-driven analysis choices.	Some concerns – Overall well-conducted but minor issues in blinding and reporting.

No – The study used validated and widely accepted tools for outcome measure ment	No – Both groups had their outcomes assessed using the same tools and procedur es.	No -The outcome assessors were explicitly stated to be blinded to group allocation, which is critical for reducing detection bias.	No - outcome s were objective , there was no opportun ity for bias due to knowled ge of group assignme nt	No - outcomes and blinded adjudicati on makes it highly unlikely that outcome assessme nt was influence d by knowledg e of the interventi on	Low risk - Outcome measure ment was appropria te and unbiased.	Yes- The data were analyzed according to a pre-specified analysis plan finalized before blinded outcome data were available. The protocol and statistical plan were published and registered (ClinicalTrials.gov NCT05668000)	Yes- The study includes multiple eligible outcome measure ments	Yes- The study conduc ted multipl e eligibl e analys es,	Low risk - Study folLow risked a registere d protocol, used clearly defined and pre-specified outcome s, and transpare ntly reported all analyses. There is no indicatio n of selective reporting or data-driven analysis choices.	Low risk – Well-conducted RCT with appropriate randomization, minimal missing data, and valid outcome measures.

No – The study used validated and widely accepted tools for outcome measure ment	No – Both groups had their outcomes assessed using the same tools and procedur es.	No -The outcome assessors were explicitly stated to be blinded to group allocation, which is critical for reducing detection bias.	No - outcome s were objective , there was no opportun ity for bias due to knowled ge of group assignme nt	No - outcomes and blinded adjudicati on makes it highly unlikely that outcome assessme nt was influence d by knowledg e of the interventi on	Low risk - Outcome measure ment was appropria te and unbiased.	Yes- Study was IRB-approved folLow risked a randomized controlled design with clearly defined primary and secondary outcomes, and used intention-to-treat analysis with pre-specified statistical methods.	Yes- The study includes multiple eligible outcome measure ments	Yes- The study conduc ted multipl e eligibl e analys es,	Low risk - Study folLow risked a registere d protocol, used clearly defined and pre-specified outcome s, and transpare ntly reported all analyses. There is no indicatio n of selective reporting or data-driven analysis choices.	Some concerns – Overall, study was well conducted but minor issues in randomization and reporting.

No – The study used validated and widely accepted tools for outcome measure ment	No – Both groups had their outcomes assessed using the same tools and procedur es.	No -The outcome assessors were explicitly stated to be blinded to group allocation, which is critical for reducing detection bias.	No - outcome s were objective , there was no opportun ity for bias due to knowled ge of group assignme nt	No - outcomes and blinded adjudicati on makes it highly unlikely that outcome assessme nt was influence d by knowledg e of the interventi on	Low risk - Outcome measure ment was appropria te and unbiased.	Yes- The data were analyzed according to a pre-specified analysis plan finalized before blinded outcome data were available. The protocol and statistical plan were published and registered (ClinicalTrials.gov ID: NCT01360203),	Yes- The study includes multiple eligible outcome measure ments	Yes- The study conduc ted multipl e eligibl e analys es,	Low risk - Study folLow risked a registere d protocol, used clearly defined and pre-specified outcome s, and transpare ntly reported all analyses. There is no indicatio n of selective reporting or data-driven analysis choices.	Some concerns – Overall, study was well conducted but minor issues in randomization and reporting.

No – The study used validated and widely accepted tools for outcome measure ment	No – Both groups had their outcomes assessed using the same tools and procedur es.	No -The outcome assessors were explicitly stated to be blinded to group allocation, which is critical for reducing detection bias.	No - outcome s were objective , there was no opportun ity for bias due to knowled ge of group assignme nt	No - outcomes and blinded adjudicati on makes it highly unlikely that outcome assessme nt was influence d by knowledg e of the interventi on	Low risk – Measure ment methods were appropria te and consistent .	Yes- The data were analyzed according to a pre-specified analysis plan finalized before blinded outcome data were available. The protocol and statistical plan were published and registered (IRCT2017010731804N1)	Yes- The study includes multiple eligible outcome measure ments	Yes- The study conduc ted multipl e eligibl e analys es,	Low risk - Study folLow risked a registere d protocol, used clearly defined and pre-specified outcome s, and transpare ntly reported all analyses. There is no indicatio n of selective reporting or data-driven analysis choices.	High risk- Randomization/ baseline balance were acceptable and outcomes used validated tools with prespecified analyses. However, outcome-depend ent missingness, open-label self-reported endpoints, and intervention performance bias

No – The study used validated and widely accepted tools for outcome measurement	No – Both groups had their outcomes assessed using the same tools and procedures.	No -The outcome assessors were explicitly stated to be blinded to group allocation, which is critical for reducing detection bias.	No - outcomes were objective, there was no opportunity for bias due to knowledge of group assignment	No - outcomes and blinded adjudication makes it highly unlikely that outcome assessment was influenced by knowledge of the intervention	Low risk. Despite lack of blinding, outcome measures were objective and standardized.	Yes- The data were analyzed according to a pre-specified analysis plan finalized before blinded outcome data were available. The protocol and statistical plan were published and registered (NCT00371085)	Yes- The study includes multiple eligible outcome measurements	Yes- The study conducted multiple eligible analyses,	Low risk - Study folLow risked a registered protocol, used clearly defined and pre-specified outcomes, and transparently reported all analyses. There is no indication of selective reporting or data-driven analysis choices.	Low risk – The study was well conducted with appropriate randomization, outcome measurement, and analysis.

No – The study used validated and widely accepted tools for outcome measurement	No – Both groups had their outcomes assessed using the same tools and procedures.	No -The outcome assessors were explicitly stated to be blinded to group allocation, which is critical for reducing detection bias.	No - outcomes were objective, there was no opportunity for bias due to knowledge of group assignment	No - outcomes and blinded adjudication makes it highly unlikely that outcome assessment was influenced by knowledge of the intervention	Low risk - Outcome measurement was appropriate and unbiased.	Yes- The data were analyzed according to a pre-specified analysis plan finalized before blinded outcome data were available. The protocol and statistical plan were published and registered (NCT01964053)	Yes- The study includes multiple eligible outcome measurements	Yes- The study conducted multiple eligible analyses,	Low risk - Study folLow risked a registered protocol, used clearly defined and pre-specified outcomes, and transparently reported all analyses. There is no indication of selective reporting or data-driven analysis choices.	Low risk- The trial had robust randomization with allocation concealment, minimal attrition, blinded outcome assessment, and adherence to a pre-specified protocol, reducing major bias risks. Minor concerns remain due to baseline imbalances and open-label design, but these are unlikely to significantly affect objective outcomes.
